# Increases in the mean and variability of thermal regimes result in differential phenotypic responses among genotypes during early ontogenetic stages of lake sturgeon (*Acipenser fulvescens*)

**DOI:** 10.1111/eva.12409

**Published:** 2016-08-31

**Authors:** Kari J. Dammerman, Juan P. Steibel, Kim T. Scribner

**Affiliations:** ^1^ Department of Integrative BiologyMichigan State UniversityEast LansingMIUSA; ^2^ U.S. Fish and Wildlife ServiceColumbia River Fish and Wildlife Conservation OfficeVancouverWAUSA; ^3^ Department of Animal ScienceMichigan State UniversityEast LansingMIUSA; ^4^ Department of Fisheries and WildlifeMichigan State UniversityEast LansingMIUSA; ^5^ Ecology, Evolutionary Biology, and Behavior ProgramMichigan State UniversityEast LansingMIUSA

**Keywords:** ectotherms, environmental change, genotype‐by‐environment interaction, lake sturgeon, phenotypic variation, temperature

## Abstract

Climate change is affecting thermal conditions worldwide. Understanding organismal responses associated with predicted changes are essential for predicting population persistence. Few studies have examined the effects of both increased mean and variance in temperature on organismal traits, particularly during early life stages. Using lake sturgeon (*Acipenser fulvescens*) from Black Lake, MI, we tested whether phenotypic variation differed among families reared in two constant (10 and 18°C) and two fluctuating‐temperature treatments (10–19°C) representing temperatures experienced in the river and a simulated anthropogenic disturbance. Body length, body area, and yolk‐sac area were quantified at hatch. Family‐by‐treatment interactions explained up to 50% of the variance observed among families in offspring hatch traits. Families incubated in 18°C and the fluctuating anthropogenic treatment had 6–10 times higher variance in traits than those incubated at 10°C. Hatched larvae were placed in raceways with ambient river water. Emergence body length, emergence timing, and growth were quantified upon emergence. Families differed in time to emergence and growth with the greatest range observed in the 18°C treatment. Results demonstrate that differential responses among genotypes to changes in the mean and variability of thermal incubation regimes can affect traits at hatch as well as a subsequent ontogenetic stage.

## 1 Introduction

Climate change is altering thermal regimes on a global scale (Intergovernmental Panel on Climate Change, 2013). Within the next century, mean temperatures are predicted to increase by approximately 2–5°C due to human influences (Estay, Lima, & Bozinovic, 2014). Additionally, increases in the magnitude and frequency of extreme climatic events are altering the variability in thermal patterns (Bauerfeind & Fischer, 2014; Pincebourde, Sanford, Casas, & Helmuth, 2012). Thermal changes are known to directly affect biological functions in wild populations. For instance, previous studies have documented advances in the timing of reproduction (Parmesan, 2006), faster growth rates (Drinkwater, 2005), and decreases in species abundance (Jonsson & Jonsson, 2009) in response to increasing temperatures. Therefore, understanding the ecological and evolutionary responses of wild populations to predicted thermal changes due to climate change has become a major goal for ecologists and climate researchers (Hansen, Olivieri, Waller, & Nielsen, 2012; Walther et al., 2002).

Ectotherms are one group of organisms being affected by climate change given that temperature directly affects their physiology including growth, reproduction, and locomotion (Deutsch et al., 2008; Jonsson & Jonsson, 2009). For example, one well‐documented trend among ectotherms is the temperature–size rule in which rearing temperature is inversely related to body size (Diamond & Kingsolver, 2010; Kingsolver & Huey, 2008). Individuals that are reared in warm temperatures grow faster, but are typically smaller than individuals reared in cold temperatures (Atkinson, 1994; Forster, Hirst, & Atkinson, 2011). Numerous studies have examined phenotypic trait changes in response to rearing temperatures in ectotherms given that changes in traits associated with fitness (i.e., body size) typically occur during early life stages which are characterized by high mortality, and are considered to be the most susceptible to thermal fluctuations (Jonsson & Jonsson, 2009). Additionally, environmental conditions experienced during early ontogeny have long‐term consequences on individual fitness by affecting developmental rates, morphology, physiology, and behavior at later ontogenetic stages (i.e., ontogenetic contingency; Diggle, 1994; Orizaola, Dahl, & Laurila, 2010; Huey et al., 2012; Crespi & Warne, 2013; Pittman et al., 2013; Dammerman, Steibel, & Scribner, 2015).

Changes in phenotypic trait expression in response to temperature change are commonly visualized as thermal reaction norms that are constructed by rearing individuals of known genotype over a range of temperatures that remain constant and quantifying the variation in phenotypes that are expressed (Angilletta, 2009). These plots have been useful for modeling the effects of climate change by exposing individuals to ecologically relevant changes in temperature and comparing their phenotypes to individuals reared under current thermal conditions in a laboratory setting. However, few studies have reared individuals under fluctuating incubation temperatures (i.e., diurnal temperature changes) that simulate the variability predicted with climate change and mimic conditions encountered in the wild (Bauerfeind & Fischer, 2014). Recent research has shown that rearing individuals under fluctuating temperatures more accurately represents responses to climate change given that organisms encounter daily fluctuations in temperature (Bauerfeind & Fischer, 2014; Niehaus, Angilletta, Sears, Franklin, & Wilson, 2012), and temperature variability is predicted to affect traits associated with fitness to a greater extent than mean temperature alone (Paaijmans et al., 2013). For example, the magnitude of daily temperature fluctuations has been shown to influence stress levels in salmonids (Thomas et al., 1986; Wehrly, Wang, & Mitro, 2007). Given that phenotypic variance often increases under stressful conditions (Ghalambor, McKay, Carroll, & Reznick, 2007), rearing individuals under fluctuating conditions may be necessary to determine how increases in temperature variability will impact wild populations.

In this study, we incubated fertilized eggs from different families of lake sturgeon (*Acipenser fulvescens*; Rafinesque 1817) under constant and fluctuating thermal incubation conditions and quantified phenotypic variation at hatch and at the time of emergence from the substrate. Lake sturgeon are long‐lived ectotherms and a threatened species in the state of Michigan that has been numerically depressed due to overharvest, habitat loss and degradation, and limited recruitment (Peterson, Vecsei, & Jennings, 2007). Rehabilitation programs that include protection of spawning and rearing habitats as well as supplemental stocking have been implemented to mitigate the effects of human activities and environmental change (Hayes & Caroffino, 2012). Lake sturgeon are a useful species for examining the effects of thermal changes given that temperature directly affects adult spawning behavior, timing of embryogenesis, and larval phenotypic traits associated with survival (Dammerman et al., 2015; Forsythe, Crossman, Bello, Baker, & Scribner, 2012).

In the spring, adults migrate to riverine areas for spawning (Auer, 1996). The timing of spawning is multimodal, and has been observed when water temperature is between 8.8 and 21.1°C (Bruch & Binkowski, 2002; Forsythe et al., 2012). During spawning events, females release demersal, adhesive eggs which are fertilized by multiple males (Thiem, Dumont, Van Der Kraak, & Cooke, 2013). Fertilized eggs from full‐ and half‐sibling families attach to the substrate and incubate without parental care under site‐specific conditions until hatch (Duong, Scribner, Crossman, Forsythe, & Baker, 2011). Larvae typically hatch within 5–14 days dependent on water temperature (Kempinger, 1988; Smith & King, 2005) and then burrow into the substrate where nourishment is provided by endogenous yolk‐sac reserves (Hastings, Bauman, Baker, & Scribner, 2013). The duration of yolk‐sac utilization until the timing of emergence from the substrate when larvae disperse downstream to begin exogenously feeding is dependent on several abiotic conditions within larval rearing sites including temperature (Duong et al., 2011). Rearing temperatures prior to exogenous feeding are known to influence the cortisol response and induce thermal stress in larval lake sturgeon (Zubair, Peake, Hare, & Anderson, 2012).

Identifying how thermal variability affects growth and survival among families during critical early life stages is an essential goal for determining the effects of climatic changes on this threatened species. Our first objective was to determine whether larval phenotypes and the timing of emergence would vary among individuals of different families when fertilized eggs were incubated in constant and fluctuating thermal environments. We predicted that phenotypic variation within the fluctuating treatments would be greater than observed under constant treatments due to a potential stress response. Additionally, we predicted that phenotypes would vary among families particularly within the fluctuating treatments given the large variance attributed to genetic (family) effects quantified in a recent study on lake sturgeon responses to stressful environmental conditions (Dammerman et al., 2015). Our second objective was to assess whether thermal environments experienced during egg incubation would affect larval growth and the timing of emergence during a subsequent ontogenetic stage. Quantifying trait changes conditional on environments experienced during previous ontogenetic stages increases understanding of how fluctuations in local thermal regimes will affect phenotypic trait variation and survival of different genotypes during critical developmental stages of ectotherms.

## 2 Materials and Methods

### 2.1 Study site

**Figure 1:**
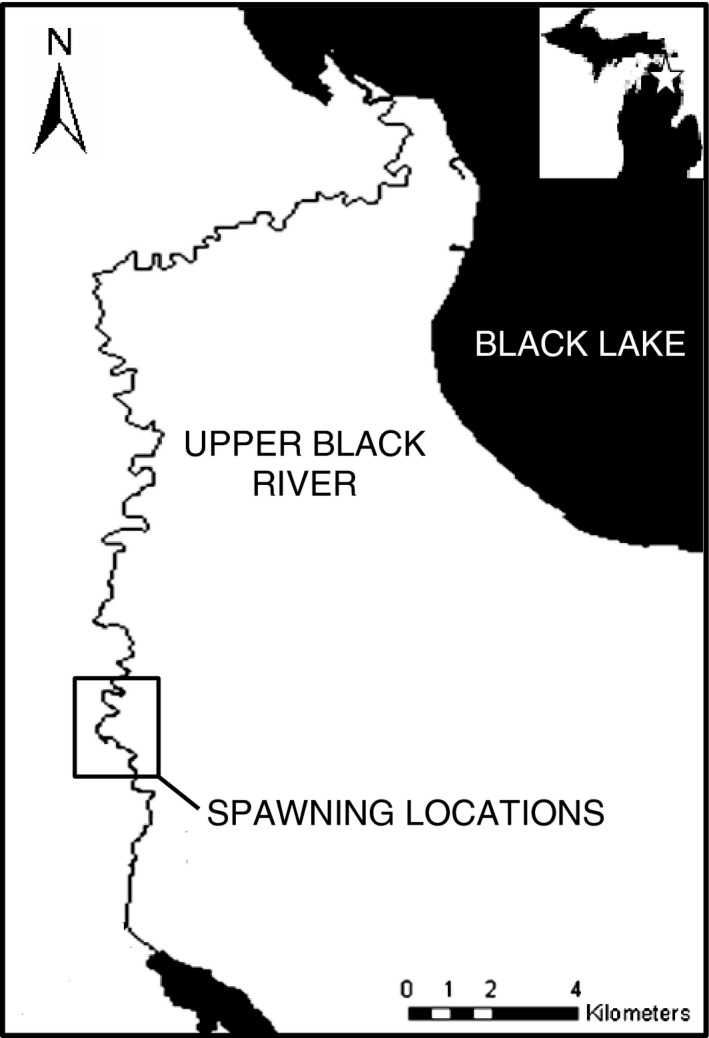
The study location on the Upper Black River, the largest tributary of Black Lake, Michigan, showing the spawning locations where adult lake sturgeon were sampled during the 2012 spawning season

During the 2012 spawning season, adult lake sturgeon were sampled daily using long‐handled dip nets on the Upper Black River (UBR). The UBR is the largest tributary of Black Lake located in Cheboygan County, Michigan (Fig. 1; Smith & King, 2005). Adults migrate into the UBR beginning in late spring to spawn among shallow (~1–3 m) rocky areas (Baker & Borgeson, 1999; Forsythe et al., 2012). Spawning activities observed early in the season (April to early May) occur when water temperature is approximately 10°C. By the later part of the season (mid‐May to June), spawning adults encounter mean water temperatures near 18°C. However, peaks in spawning activity have been observed over a range of temperatures due to the interannual variability in temperature (Forsythe et al., 2012). The wadable conditions of the UBR and the presence of a streamside rearing facility provide the opportunity to collect gametes from spawning adults and conduct experimental temperature manipulations and monitoring of larval traits.

### 2.2 Fertilizations

Gametes were collected from “early” spawning fish during the first major peak in spawning on May 3, 4, and 6, 2012, when mean water temperature was approximately 13°C. Eggs were taken from five spawning females, placed in sealed plastic bags, and stored with river water to maintain eggs at ambient river temperature. Milt was collected from ten spawning males using 20‐ml syringes and placed on ice. Gametes were transported to the streamside rearing facility, and fertilizations were conducted within twelve hours of collection. Approximately 1,600 eggs were collected from each female and placed on 1‐mm mesh screens within eight polymerized vinyl chloride couplings (31.90 cm^2^) used to keep families separate. Each female's eggs were fertilized with 0.5 ml of milt from two males to create ten half‐sibling (HS) families where all ten males were represented and no males were used more than once. Fertilized eggs were widely distributed within the couplings to prevent them from touching and left undisturbed for 30 min to allow adhesion to the mesh screens.

### 2.3 Thermal incubation treatments and traits measured at hatch

**Figure 2:**
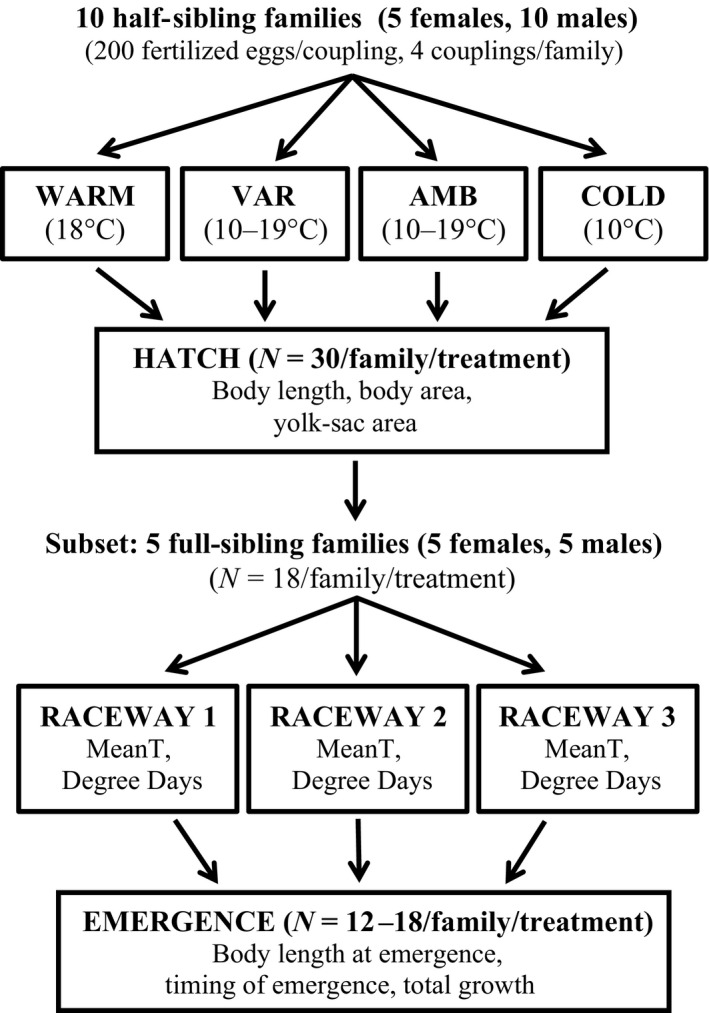
Diagram of the experimental design used in the study. Fertilized eggs from 10 half‐sibling families were incubated in four thermal treatments: warm, variable (Var), ambient (Amb), and cold until hatch. After being photographed at hatch to quantify traits, larvae were subset from five full‐sibling families to track the effect of incubation conditions on traits at a subsequent ontogenetic stage. Subset larvae were placed in chambers within three raceways with ambient river water where the mean temperature (Mean T) and degree‐days (Degree Days) experienced while in the chambers were recorded for each individual. Individuals were photographed again at emergence to quantify traits

**Figure 3:**
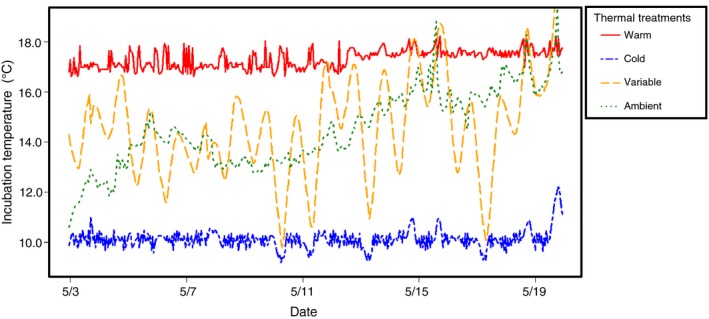
The four egg incubation temperatures (warm, cold, variable, and ambient) in which individuals were incubated in during the 2012 spawning season

Fertilized eggs were placed in vertical incubators where water flows from top to bottom (i.e., heath trays) where temperature was controlled using heating and cooling units to produce two constant and two fluctuating thermal treatments during the egg incubation period (Fig. 2). Constant treatments, cold (10°C) and warm (~18°C), represented mean temperatures that adults encounter in the UBR during the early‐ and late‐season spawning periods, respectively (Forsythe et al., 2012), as well as the range in temperature where spawning activities have been observed in lake sturgeon (Bruch & Binkowski, 2002) across the species' native range. River water from the stream was pumped through the heath trays to produce the ambient treatment where temperatures fluctuated naturally (approximately 1–3°/day). The variable treatment simulated an anthropogenic disturbance with a range of 1–9 degrees of change per day. This range was chosen to determine the effect of rapid diel changes in temperature (5–10°) that have been observed on the UBR due to earlier spring thaw and loss of ice cover on reservoirs as well as excessive precipitation leading to flooding events (data not shown). These environmental extremes are predicted to increase in magnitude and frequency throughout the entire Great Lakes region due to climate change (Patz, Vavrus, Uejio, & McLellan, 2008) thereby impacting numerous species including lake sturgeon. Water temperature within each treatment was measured daily during the egg incubation period (Fig. 3) using Onset HOBO pressure loggers (Cape Cod, MA, USA). Fertilized eggs were slowly acclimated to treatment temperatures (two‐degree change per hour) prior to placement in heath trays. Families were replicated among incubation treatments and dead eggs were removed daily to prevent fungal infections. Egg mortality for each family was not recorded due to the inability to differentiate between the lack of fertilization or mortality due to natural causes.

Thirty larvae from each family within each thermal incubation treatment (*N* = 1,200 total) were removed from the heath trays, anesthetized with tricaine methanesulfonate (MS‐222; 25 mg/ml), and photographed using a digital camera and a ruler. Photographs were analyzed using ImageJ analysis software (version 1.34, freeware) to quantify three phenotypic traits at hatch: body length (mm), body area (mm^2^), and yolk‐sac area (mm^2^; Dammerman et al., 2015).

### 2.4 Rearing chambers and traits measured at the timing of emergence

To determine whether incubation conditions affected traits at a subsequent ontogenetic stage, we monitored a subset of larvae from each of the thermal incubation treatments until the time of emergence from the substrate. Due to space restrictions, eighteen individuals from five of the ten HS families were subsampled from each of the thermal incubation treatments (Fig. 2; *N* = 360 total) allowing offspring from all females to be represented, but only five males from the fertilization scheme. Larvae that were subset and monitored until emergence were unrelated (i.e., full‐sibling design); therefore, we refer to them as full‐sibling (FS) families. Each individual larva that was subsampled from the five FS families was randomly assigned to an individual plastic rearing chamber (12.7 cm by 6.35 cm). Rearing chambers contained only one fish, gravel substrate to provide cover during the yolk‐sac utilization period, and had mesh siding to allow for continual water passage (~0.1 m/s). Chambers were randomly placed across three fiberglass raceways (3.7 m by 0.67 m; Fig. 2) with respect to family and thermal incubation treatment to prevent any influence of chamber location on the timing of emergence. Ambient river water from the stream was pumped into each of the three raceways to allow larvae to experience similar conditions while in the chambers. Rearing chambers were monitored daily approximately every four hours. Larvae with eyes, gills, barbels, and translucent pectoral fins that swam out of the substrate to the top of the chambers and had no visible signs of a residual yolk‐sac reserve were designated as emerged larvae. Once individuals emerged from the substrate to begin exogenously feeding, larvae were anesthetized and photographed again to quantify body length at emergence (mm), growth from hatch to emergence (mm), and the time from hatch to emergence (days).

Prior to the start of the experiment, we predicted that individuals within the thermal incubation treatments would hatch on different days due to the well‐known relationship between embryogenesis and incubation temperature in ectotherms (Kingsolver & Huey, 2008). Therefore, hatched larvae were unavoidably expected to experience subtle differences in ambient stream temperatures during yolk‐sac utilization in the rearing chambers. Given that yolk‐sac utilization and timing of emergence are temperature‐dependent in larval fishes including sturgeon (Duong et al., 2011; Hardy & Litvak, 2004), we tested the effect of the ambient river temperatures within the raceways on the three traits measured at the time of emergence. Temperature data during the yolk‐sac utilization period were collected hourly for the entire streamside facility using a YSI 5200 Recirculating System Monitor (Xylem, Inc.). For each individual, we recorded the times and dates they were present within the chambers allowing us to calculate the overall mean temperature (Mean *T*) and cumulative degree‐days (Degree Days) each larva experienced while in the rearing chambers. Degree Days was estimated as a sum of the time (or amount of thermal energy) spent at a daily temperature above the threshold temperature, *T*_0_ (Chezik, Lester, & Venturelli, 2014). We set the threshold temperature to 0°C in our calculations given that lake sturgeon growth is essentially zero at that temperature.

All research was conducted under animal use and care procedures approved by the Michigan State University Institutional Animal Care and Use Committee.

### 2.5 Statistical analysis

Statistical analyses were performed using the program R (version 3.1.2; R Development Core Team) and the Bayesian inference package MCMCglmm (Hadfield, 2010). Linear mixed‐effects models were used to test the contribution of family, incubation treatment, and a family‐by‐treatment interaction on the six larval traits measured in the study. All models included a burn‐in of 10,000, thinning interval of 100, and a total of 400,000 iterations which were chosen after examining the shape and range of values of the trace plots (Data S1) and autocorrelation plots to determine model convergence, and running Heidelberger and Welch's convergence diagnostic tests. Variances associated with the random effects including the heterogeneous residual (error) variances and the interaction term were fit using relatively uninformative priors as discussed in Hadfield (2014). The variances attributed to HS (or FS) Family were given an inverse‐gamma distribution with a scale and shape of 0.001 which results in a noninformative prior. A sensitivity analysis conducted by fitting models with a proper prior attributing a higher degree of the phenotypic variance to genetic effects revealed there was little effect of the priors on the posterior distribution.

Model selection was based on the deviance information criterion (DIC; Table S1) which incorporates model fit and complexity based on an expected deviance parameter and the effective number of parameters estimated in the model (Spiegelhalter, Best, Carlin, & van der Linde, 2002). We compared DIC estimates for the most complex model against all possible simpler models (Table S1). A stepwise approach was used to allow us to eliminate the variables of the least biological meaning or those that only accounted for possible experimental errors in an ordered fashion. While generally the fixed effects must remain constant among models being compared when models are fit using REML, a Bayesian approach allowed us to remove fixed effects during the selection process if the 95% credible interval profiled from the posterior distributions spanned zero (Wilson et al., 2010). When models were within two DIC values of each other and visually comparable in fit, the simplest model was chosen as the best model (Spiegelhalter et al., 2002). Models of best fit without the interaction term were also compared to a simpler model with homogeneous residual error variance. Parameter estimates were calculated as the modes of the posterior distributions estimated from the model of best fit. The 95% highest posterior density (HPD) was also estimated for each parameter.

The three traits measured at hatch were analyzed separately by fitting the mode l:

(1) $$\begin{array}{cc} \text{Trait}\;(y_{ijk})= & \mu +{\text{Incubation Treatment}}_{i}+{\text{HS Family}}_{j}\\ & +{\left(\text{HS Family}\times \text{Incubation Treatment}\right)}_{ij}+\varepsilon_{ijk} \end{array}$$

where μ is the population mean, (*x*) represents an interaction between variables, and ε_*ijk*_ represents the random heterogeneous residual errors which follow Gaussian distributions. Incubation treatment was fit as a fixed effect. HS Family and the HS Family‐by‐Incubation Treatment interaction terms were fit as random effects with ${\text{HS Family}}_{j}∼N\;\left(0,\sigma_{F}^{2}\right),\;{\left(\text{HS Family}\times \text{Incubation Treatment}\right)}_{ij}∼N\;\left(0,\sigma_{FS}^{2}\right)$, and $\varepsilon_{ijk}∼N\;\left(0,\sigma_{\varepsilon }^{2}\right)$. The across‐treatment covariances in family effects were set to zero. Preliminary analyses included fitting full models where covariances were estimated using the us function, but these complex models had comparable trace plots and DIC estimates (within ± 2) as simpler models with covariances set to zero using the idh function. Therefore, we used the simpler models in all of our analyses. All models contained HS family as a random effect as opposed to fitting an animal‐specific random effect (i.e., animal model; Wilson et al., 2010). Preliminary analyses indicated that parameter estimates were comparable using either model, but the animal model had convergence issues indicating an inappropriate fit for the data. Our study contained a simple pedigree with large sibling groups and a genotype‐by‐environment interaction which can cause biases (due to dominance effects) and convergence issues when fitting an animal model. Therefore, models were parameterized with the simpler HS (or FS) family effect model which was comparable and simpler to implement.

The three traits quantified at the time of emergence were analyzed in a similar manner with the addition of Raceway, Mean *T*, and Degree Days experienced during the yolk‐sac utilization period within the rearing chambers as fixed effects. We included the incubation treatment experienced during the egg stage and a FS Family‐by‐Incubation Treatment interaction within the model to determine whether conditions experienced during the egg incubation stage affect larval traits at a later ontogenetic stage. Therefore, the full model for the traits measured at emergence was:

(2) $$\begin{array}{cc} \text{Trait}\;(y_{ijk})= & \mu +{\text{Incubation Treatment}}_{i}+\text{Mean}\;T_{j}\\ & +{\text{Degree Days}}_{k}+{\text{Raceway}}_{l}+{\text{FS Family}}_{m}\\ & +{\left(\text{FS Family}\times \text{Incubation Treatment}\right)}_{im}+\varepsilon_{ijklm} \end{array}$$

The correlation between Mean *T* and Degree Days was low (Pearson's correlation = 0.16, *p* < 0.001) allowing us to test the variables in the same model.

For traits where the model of best fit included a FS Family effect but no FS Family‐by‐Incubation Treatment interaction, the mode of the posterior distribution for heritability in the broad‐sense (*H* ^2^) and 95% HPD intervals were computed (Table 2). Heritability was estimated to determine what fraction of the variance in the phenotypic traits observed among individuals was due to their genotypes, and provide insight into potential changes in the traits in response to environmental conditions. Heritability (*H* ^2^) was estimated as two times the FS Family variance divided by the total phenotypic variance equivalent to the heritability estimates derived in Dammerman et al., 2015 (see appendix 1). Estimates were treated as heritability in the broad‐sense (*H* ^2^) or the upper limit of a narrow‐sense heritability given that we were unable to separate out additive genetic variance from other genetic effects. The predict function was used to produce plots of the conditioned means and the 95% prediction intervals within each of the four thermal incubation treatments. The predict function is often used to estimate new values from the fitted model which are then compared to the actual values to determine model fit. In our study, we plot the conditional means using the predict function to determine the effect of our fixed and random effects in the study.

To empirically determine whether variances among families differed among treatments, we created a profile of *F*‐ratios which were obtained by extracting the posterior distributions for each of the FS Family‐by‐Incubation Treatment terms from the model of best fit. The numerator variance (variance 1) was expected to be larger than the denominator variance (variance 2) resulting in a one‐tailed test. An empirical tail‐area probability from the *F*‐ratios was calculated as one minus the sum of the ratios that were greater than one divided by the total number of *F*‐ratios (Table 2). For each of the variances that were compared, the total number of *F*‐ratios was 3,900 given that there were 3,900 iterations for each of the posterior distributions in the models of best fit.

## 3 Results

**Figure 4:**
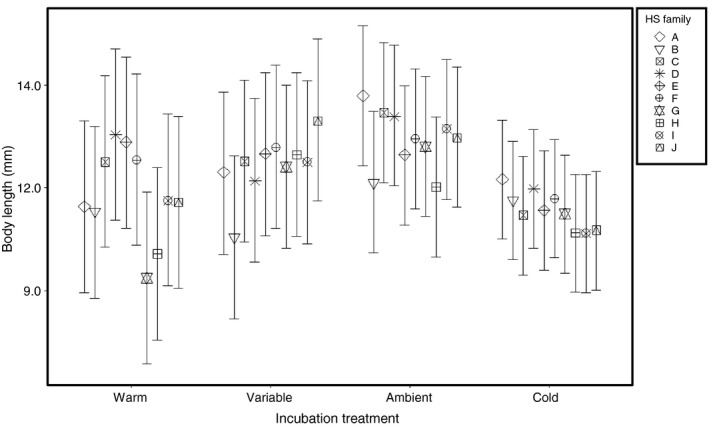
Thermal reaction norm plot including the conditioned family means ± 95% prediction intervals for larval body length at hatch for 10 half‐sibling (HS) lake sturgeon families reared in the four thermal incubation treatments

Larvae began to hatch from the warm thermal treatment beginning May 9th, approximately six days postfertilization. Half‐sibling (HS) families showed considerable variation in the three traits measured at hatch across the four treatments. Mean body length (±*SE*) ranged from 10.19 (±0.09) to 13.84 (±0.09) mm among families (Fig. 4). Among HS families, body length varied due to a significant HS Family‐by‐Incubation Treatment interaction (Table S1) which explained approximately 49% of the phenotypic variation observed. Family variances estimated within each treatment ranged from 0.18 to 1.10 (Table 1), and were different except when comparing the ambient versus variable treatments (Table 2). Families reared in the warm treatment had the greatest variance, and up to six times higher variance than families reared in the cold treatment (Table 1).

**Table 1:** Variance components and 95% highest posterior density (HPD) estimated from the models of best fit for the three traits measured at hatch (a) and three traits measured at the time of emergence (b). Thermal treatments are denoted as warm (Warm), variable (Var), ambient (Amb), and cold (Cold). Heritability (H2) estimates are provided for traits where the model of best fit included a significant half‐sibling (HS) or full‐sibling (FS) Family effect, but no Family‐by‐Incubation Treatment interaction

Phenotypic traits	Component	Var	±95% HPD
(a) Measured at hatch			
Body length	HS Family*Warm	1.10	(0.27, 2.41)
	HS Family*Var	0.46	(0.12, 1.02)
	HS Family*Amb	0.44	(0.12, 0.94)
	HS Family*Cold	0.18	(0.04, 0.40)
	Residual_Warm_	0.74	(0.62, 0.85)
	Residual_Var_	0.66	(0.56, 0.77)
	Residual_Amb_	0.49	(0.42, 0.57)
	Residual_Cold_	0.35	(0.30, 0.41)
Body area	HS Family*Warm	9.27	(2.15, 20.44)
	HS Family*Var	10.53	(2.84, 22.65)
	HS Family*Amb	4.73	(1.10, 10.32)
	HS Family*Cold	1.21	(0.22, 2.71)
	Residual_Warm_	6.00	(5.07, 7.01)
	Residual_Var_	9.01	(7.57, 10.44)
	Residual_Amb_	8.45	(7.11, 9.87)
	Residual_Cold_	2.64	(2.24, 3.09)
Yolk‐sac area	HS Family*Warm	0.10	(0.02, 0.24)
	HS Family*Var	0.32	(0.08, 0.70)
	HS Family*Amb	0.05	(<0.01, 0.14)
	HS Family*Cold	0.03	(<0.01, 0.07)
	Residual_Warm_	0.45	(0.38, 0.53)
	Residual_Var_	0.52	(0.44, 0.61)
	Residual_Amb_	0.75	(0.63, 0.88)
	Residual_Cold_	0.40	(0.34, 0.47)
(b) Measured at the time of emergence			
Time to emergence	FS Family	0.36	(<0.01, 1.17)
	Residual_homogeneous_	2.69	(2.25, 3.11)
	*H* ^2^	0.21	(<0.01, 0.61)
Emergence body length	Residual_homogeneous_	2.38	(2.01, 2.77)
Total growth	FS Family	0.59	(0.01, 1.77)
	Residual_homogeneous_	2.47	(2.08, 2.88)
	*H* ^2^	0.32	(0.02, 0.85)

*Indicates an interaction between the components.

**Table 2:** Results of the one‐tailed *F*‐ratio tests comparing the half‐sibling family (HSF) variances across treatments extracted from the models of best fit. Thermal treatments are denoted as warm (Warm), variable (Var), ambient (Amb), and cold (Cold). the numerator variance (Variance1) was expected to be larger than the denominator variance (Variance2). The empirical tail area probability was calculated as one minus the sum of the *F*‐ratios greater than one (*N*) divided by the total number of *F*‐ratios (3,900)

Phenotypic traits	Variance_1_	Variance_2_	*N*	Tail‐area probability
Body length	$\sigma_{\mathrm{HSF}*\mathrm{Warm}}^{2}$	$\sigma_{\mathrm{HSF}*\mathrm{Var}}^{2}$	3,441	0.118^a^
	$\sigma_{\mathrm{HSF}*\mathrm{Warm}}^{2}$	$\sigma_{\mathrm{HSF}*\mathrm{Amb}}^{2}$	3,507	0.101^a^
	$\sigma_{\mathrm{HSF}*\mathrm{Warm}}^{2}$	$\sigma_{\mathrm{HSF}*\mathrm{Cold}}^{2}$	3,868	0.008^a^
	$\sigma_{\mathrm{HSF}*\mathrm{Var}}^{2}$	$\sigma_{\mathrm{HSF}*\mathrm{Amb}}^{2}$	2,040	0.477
	$\sigma_{\mathrm{HSF}*\mathrm{Var}}^{2}$	$\sigma_{\mathrm{HSF}*\mathrm{Cold}}^{2}$	3,487	0.106^a^
	$\sigma_{\mathrm{HSF}*\mathrm{Amb}}^{2}$	$\sigma_{\mathrm{HSF}*\mathrm{Cold}}^{2}$	3,461	0.113^a^
Body area	$\sigma_{\mathrm{HSF}*\mathrm{Var}}^{2}$	$\sigma_{\mathrm{HSF}*\mathrm{Warm}}^{2}$	2,291	0.413
	$\sigma_{\mathrm{HSF}*\mathrm{Warm}}^{2}$	$\sigma_{\mathrm{HSF}*\mathrm{Amb}}^{2}$	3,222	0.174^a^
	$\sigma_{\mathrm{HSF}*\mathrm{Warm}}^{2}$	$\sigma_{\mathrm{HSF}*\mathrm{Cold}}^{2}$	3,883	0.004^a^
	$\sigma_{\mathrm{HSF}*\mathrm{Var}}^{2}$	$\sigma_{\mathrm{HSF}*\mathrm{Amb}}^{2}$	3,354	0.140^a^
	$\sigma_{\mathrm{HSF}*\mathrm{Var}}^{2}$	$\sigma_{\mathrm{HSF}*\mathrm{Cold}}^{2}$	3,893	0.002^a^
	$\sigma_{\mathrm{HSF}*\mathrm{Amb}}^{2}$	$\sigma_{\mathrm{HSF}*\mathrm{Cold}}^{2}$	3,761	0.036^a^
Yolk‐sac area	$\sigma_{\mathrm{HSF}*\mathrm{Var}}^{2}$	$\sigma_{\mathrm{HSF}*\mathrm{Warm}}^{2}$	3,635	0.068^a^
	$\sigma_{\mathrm{HSF}*\mathrm{Warm}}^{2}$	$\sigma_{\mathrm{HSF}*\mathrm{Amb}}^{2}$	3,024	0.225^a^
	$\sigma_{\mathrm{HSF}*\mathrm{Warm}}^{2}$	$\sigma_{\mathrm{HSF}*\mathrm{Cold}}^{2}$	3,578	0.083^a^
	$\sigma_{\mathrm{HSF}*\mathrm{Var}}^{2}$	$\sigma_{\mathrm{HSF}*\mathrm{Amb}}^{2}$	3,821	0.020^a^
	$\sigma_{\mathrm{HSF}*\mathrm{Var}}^{2}$	$\sigma_{\mathrm{HSF}*\mathrm{Cold}}^{2}$	3,889	0.003^a^
	$\sigma_{\mathrm{HSF}*\mathrm{Amb}}^{2}$	$\sigma_{\mathrm{HSF}*\mathrm{Cold}}^{2}$	2,670	0.315

a Indicates a suggested difference between the variance components.

Mean body area and mean yolk‐sac area at hatch also varied significantly among HS families across treatments ranging from 16.82 (±0.17) to 28.46 (±0.32) mm^2^ and 6.91 (±0.11) to 8.11 (±0.14) mm^2^, respectively (Figs S2 and S3). Families reared in the variable treatment had nine to ten times higher variance in the two traits than families reared in the cold treatment (Table 1). Additionally, a majority of the pairwise *F*‐ratio tests comparing the family variances estimated within each of the four treatments were different for both traits (Table 2). HS Family‐by‐Incubation Treatment interactions were significant for both body area and yolk‐sac area, explaining approximately 50% and 19% of the variation, respectively.

**Figure 5:**
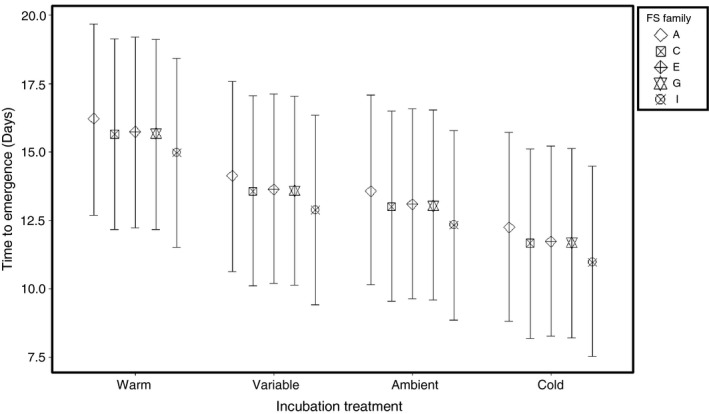
Thermal reaction norm plot for the timing to emergence including the conditioned family means ± 95% prediction intervals for the five full‐sibling (FS) families

Approximately 87% of the larvae from the five subsampled, full‐sibling (FS) families placed in the rearing chambers emerged from the substrate to begin exogenously feeding. Individuals began to emerge 10 days after being placed in the chambers. FS families differed in the mean time spent in the rearing chambers ranging from 12.67 (±0.34) to 14.21 (±0.26) days (Fig. 5) with fish from the warm incubation treatment spending approximately 2–4 days on average longer in the chambers (Table S2). Degree Days experienced while in the raceways had a slight effect on the mean timing of emergence, but the variance observed among individuals was due to a FS Family effect (Table S1). The heritability (*H* ^2^ ± 95% HPD interval) for emergence timing was estimated as 0.21 (<0.01, 0.61; Table 1). No FS Family‐by‐Incubation Treatment interaction, Raceway effect, or effect of mean temperatures experienced while in the chambers (Mean *T*) was detected for the timing of emergence (Table S1).

**Figure 6:**
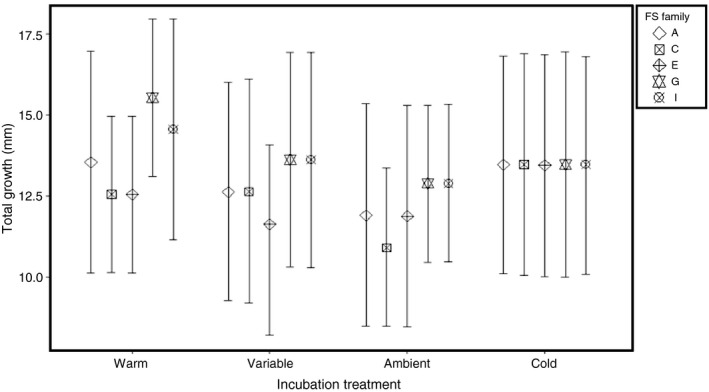
Conditioned family means** **± 95% prediction intervals for larval lake sturgeon growth measured from hatch to the timing of emergence in the five full‐sibling (FS) families

Total growth from hatch to emergence differed among FS families ranging from 10.97 (±0.35) to 15.39 (±0.47) mm (Fig. 6). Fish reared in the warm thermal treatment grew on average 1.00 mm larger than larvae in the two fluctuating treatments, but had essentially the same mean growth as fish reared in the cold treatment (Table S2). Within the warm treatment, FS families differed in mean growth by approximately 2.70 mm as opposed to fish in the cold treatment where mean body size among FS families differed by less than 1.00 mm. Variability in growth among larvae was attributed to a FS Family effect (Table S1) where the mode and 95% HPD of the *H* ^2^ distribution were estimated as 0.32 (0.02, 0.85; Table 1).

**Figure 7:**
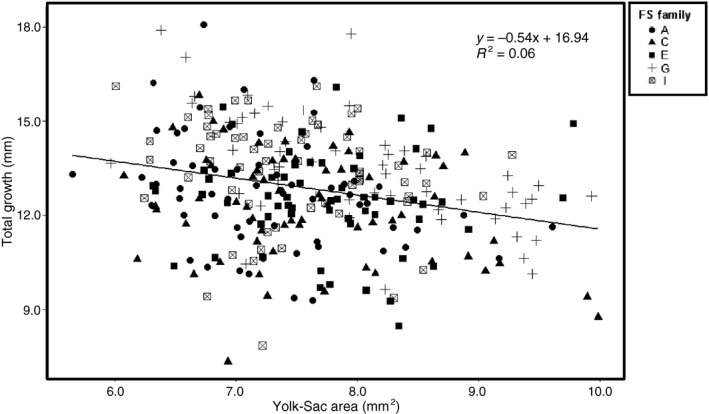
Plot showing the relationship between yolk‐sac area and total growth (*R* ^2^ = 0.06) for the five full‐sibling (FS) families monitored from hatch until emergence

Given that maternal effects can influence growth, we plot the maternally provisioned yolk‐sac area versus total growth for larvae from the five subsampled FS families. Using a linear regression, yolk‐sac area was a significant addition to the model (*p* < 0.01), but weakly explained the variability observed in growth from hatch to emergence (*R* ^2^ = 0.06; Fig. 7). Additionally, no FS Family‐by‐Incubation Treatment interaction, Raceway effect, or effect of temperatures experienced while in the rearing chambers (Mean *T* and Degree Days) was detected for total growth (Table S1). Variation in mean body size at the timing of emergence was low among FS families ranging from 24.57 (±0.20) to 25.45 (±0.16) mm, and no FS Family, FS Family‐by‐Incubation Treatment, Incubation Treatment, Raceway effect, or effect of temperatures experienced while in the rearing chambers (Mean *T* and Degree Days) was detected (Table S1 and Fig. S5).

## 4 Discussion

The early life stages of many ectothermic species are characterized by high levels of mortality (Fuiman & Werner, 2002; Vitt & Caldwell, 2014). Increased temperatures encountered during early ontogeny are known to affect phenotypic traits in ectothermic species (Angilletta, Niewiarowski, Dunham, Leaché, & Porter, 2004; Atkinson, 1996). However, there is a limited understanding of how thermal variability during egg incubation will affect trait variation at hatch and during sequential ontogenetic stages. Empirical work quantifying the effects of both increased mean and variability in temperature is necessary to predict the ecological and evolutionary responses of populations to future climatic regimes. In this study, we experimentally manipulated egg incubation temperatures in a common garden experiment and demonstrated large effects of thermal regimes, genetic (family) effects, and their interaction on phenotypic trait variation during consecutive early life stages. Results demonstrate that rearing families under constant incubation temperatures does not lead to the same degree of phenotypic variation that was observed when rearing families under fluctuating temperatures. Additionally, trait differences among families exposed to different incubation conditions that were observed at hatch persisted to a subsequent ontogenetic stage (i.e., time of emergence).

### 4.1 Incubation temperatures influence phenotypic variation observed at hatch

Temperature during the egg incubation period had a significant effect on the phenotypic variation observed among HS families in the traits measured at hatch. Larvae from HS families that experienced the warm (18°C) egg incubation treatment had the largest range in mean body length at hatch (Fig. 4) and largest family variance in comparison with the other treatments (Table 1). Although lake sturgeon have been observed spawning in temperatures up to 21.1°C (Bruch & Binkowski, 2002), the gametes used in our experiment were taken from adults that typically spawn during the early part of the season when water temperatures are closer to 10°C. Therefore, the large range in mean body length and increased variance observed in the warm thermal treatment may be due to a stress‐induced plasticity associated with the increased temperature. Both genetic and phenotypic variances can increase in stressful environments due to release of cryptic genetic variation (Ghalambor et al., 2007) which is not commonly expressed under conditions that are typically encountered (Badyaev, 2005; Ledón‐Rettig, Pfennig, Chunco, & Dworkin, 2014). Thus, our results indicate that differential responses among genotypes to mean thermal regimes may increase phenotypic variation within the population thereby increasing the likelihood of exposing variant phenotypes to selection.

The presence of a genotype‐by‐environment interaction can be indicative of the release of cryptic variation (Paaby & Rockman, 2014; Schlichting, 2008). In our common garden experiments, HS Family‐by‐Incubation Treatment interactions were detected for body length, body area, and yolk‐sac area measured at hatch. The presence of the HS Family‐by‐Incubation Treatment interactions indicates that families were responding differently to the same thermal incubation environments. A large proportion of the observed phenotypic variation in the traits (~19%–50%) was explained by the HS Family‐by‐Incubation Treatment interaction. Comparably, other studies (Beacham, 1988, 1990) also found that genotype‐by‐temperature interactions explained approximately 5%–49% in morphometric traits in *Oncorhynchus gorbuscha* and *Oncorhynchus keta* after rearing individuals under different thermal regimes. These results indicate that water temperature can have a significant effect on the variability of phenotypes expressed during early development.

During model selection, it was empirically determined that the across‐treatment covariances in HS Family effects were essentially zero. Therefore, covariances were set to zero using the idh function in all of the analyses. The lack of across‐treatment covariances in the family effects indicates that families with high mean trait values in one thermal treatment did not necessarily have high trait values in the other treatments. Therefore, the putative release of cryptic genetic variation can lead to increased variability in phenotypic traits at hatch which may lead to different survival among genotypes if selection favors larger individuals; however, which families will have high or low phenotypic trait values and potentially be favored by selection is unpredictable in advance. Therefore, consideration of parentage, interaction effects, and cryptic genetic variation is important to understand how predicted thermal changes will affect phenotypic variation within wild populations.

### 4.2 Fluctuating and increasing temperatures experienced during egg incubation influence the range of phenotypic variation observed in larval traits

In the ambient incubation treatment where temperatures fluctuated naturally throughout the day, the HS Family variances estimated for body area and yolk‐sac area were much larger than those observed in the constant cold treatment (Table 1). Additionally, HS Family variances within the variable treatment were approximately 9–10 times larger than the HS Family variances observed in the constant cold treatment (Table 1). In comparison with the warm treatment, the HS Family variances for body area and yolk‐sac area estimated in the variable treatment were larger, but variances observed for the warm treatment were also much larger than those observed in the cold treatment (Table 1). Collectively, results support previous research in that incubating individuals at a constant temperature reflecting the mean thermal regime observed in the wild at that time will not always accurately represent the extent of phenotypic variation that would be observed and on which selection would act on in wild‐reared larvae when even modest daily temperature fluctuations occur (Bauerfeind & Fischer, 2014; Niehaus et al., 2012). Similarly, Niehaus et al. (2012) found that growth and developmental rates of striped marsh frogs (*Limnodynastes peronii*) reared in fluctuating thermal treatments were continually underpredicted based on reaction norms constructed from individuals reared at constant rearing temperatures. Therefore, rearing individuals under fluctuating treatments provides a more accurate representation of phenotypic trait variance and any potential genotype‐by‐environment interactions that might occur in the wild in response to climatic changes.

Within the fluctuating treatments, we also observed that the HS Family variance within the variable treatment representing the anthropogenic disturbance was 2–6 times larger for body area and yolk‐sac area than observed under “natural” conditions in the ambient treatment (Table 1). The increased variance observed within the variable treatment may also be due to a stress response as observed in the warm treatment given that the lake sturgeon population on the UBR does not experience such large diel fluctuations on a continual basis. Collectively, results suggest that increases in both the mean and variability of temperature regimes have the potential to alter phenotypic variation within sturgeon and potentially other wild populations.

### 4.3 Temperatures experienced during egg incubation affect trait variation at a subsequent ontogenetic stage

Thermal conditions experienced during egg incubation are known to affect phenotypic traits associated with survival such as shape, color, behavior, and size at developmental stages beyond hatch in several taxa of ectotherms (e.g., fishes: Martell, Kieffer, & Trippel, 2005; reptiles: Goodman, 2008; amphibians: Orizaola et al., 2010). In our study, families incubated in the fluctuating thermal treatments showed lower mean growth from hatch to emergence than those reared in the constant treatments indicating that egg incubation temperature affects trait expression at a subsequent ontogenetic stage. Additionally, families within the warm treatment show the greatest range in mean growth (Fig. S6), indicating that the size differentials observed among families at hatch are persisting until emergence. The persistence of the large range in mean growth in the warm treatment suggests that thermally‐induced plasticity could potentially be maintained across ontogenetic stages.

During early life stages, body size is associated with survival as larger individuals typically have lower levels of mortality (Brown & Shine, 2004; Fischer, Taborsky, & Kokko, 2011; Perez & Munch, 2011). Therefore, differential growth among families may lead to changes in the genetic composition of the population if selection favors genotypes that produce larger offspring during critical development periods. In our study, we are limited in our ability to predict the accommodation of thermally‐induced phenotypes (i.e., whether phenotypic variants can survive and eventually reproduce in the population) given that we only measured individuals to emergence and that the long generation time of lake sturgeon requires long‐term monitoring. However, maladaptive phenotypic variants are typically eliminated by selection (Ghalambor et al., 2007), suggesting that variation in growth among genotypes can lead to differential, family‐specific survival.

Several abiotic and biotic factors affect the timing of transition between early ontogenetic stages (Day & Rowe, 2002). In larval fishes, the timing of emergence is typically under stabilizing selection (Crozier et al., 2008) and can be dependent on heritable variation, maternal effects, and/or environmental conditions such as temperature (Curry, Noakes, & Morgan, 1995; Einum & Fleming, 2000; Skoglund, Einum, & Robertsen, 2011). In our experiment, larvae that were reared in the warm thermal treatment during egg incubation spent longer in the rearing chambers before emerging. Although larvae from the warm treatment hatched first, they were placed in the raceways when the ambient river water may have been slightly cooler thus slowing their development. The ambient river water experienced while in the chambers had a slight effect on the timing of emergence for all larvae given that Degree Days was a significant component in the model of best fit. However, approximately 21% of the variance observed among larvae in the timing of emergence was attributed to a FS Family effect which is consistent with a study on salmonids where families emerged at different times due to differential responses to thermal variation experienced during incubation (Steel et al., 2012). Our estimated heritability for the timing of emergence was *H* ^2^ = 0.21 (<0.01, 0.61). Other studies have reported narrow‐sense heritability (*h* ^2^) estimates of 0.15–0.20 for emergence timing in fishes (Carlson & Seamons, 2008; Chervet, Zöttl, Schürch, Taborsky, & Heg, 2011) indicating that additive genetic effects can have a considerable role on the phenotypes being expressed during early fish development. However, we were limited in the study on making strong inferences based on our broad‐sense estimates given that they are confounded by additional genetic effects, and they were obtained from a modest number of families. Precise heritability estimates require a large number of observations (Visscher, Hill, & Wray, 2008). Therefore, our estimates can only provide insight into the potential ability of the population to respond genetically (i.e., changes in gene frequency) to changes in thermal regimes.

### 4.4 Family differences in yolk‐sac quality and utilization may affect growth rates

Differences in maternal provisioning of endogenous yolk‐sac reserves have been well documented in ectotherms where offspring with larger yolk reserves typically have higher survival and grow to a larger body size (Dziminski & Roberts, 2006; Gagliano & McCormick, 2007; Kamler, 2005). In our study, there was no relationship between yolk‐sac area at hatch and total growth from hatch to emergence for the five FS families monitored until emergence (*R* ^2^ = 0.06; Fig. 7). However, there was differential growth among FS families suggesting that initial differences among genotypes at hatch are being maintained to the timing of emergence. Additionally, the lack of a difference in body size at the time of emergence was consistent with the results reported by Steel et al. (2012) in salmonids where thermal variance during incubation did not affect fish length at the time of emergence. Our results suggest that larvae that were smaller at hatch may have found some way to compensate while in the chambers and emerge at roughly the same size as larvae that were larger at hatch. One explanation is that larvae may have differed in the quality and utilization of their endogenous yolk‐sac reserves. Yolk‐sacs contain maternally allocated carbohydrates, proteins, and lipids which larvae absorb during the yolk‐sac utilization period (Kamler, 2008). Comparisons between two sturgeon species documented that smaller larvae were more efficient at utilizing yolk‐sac reserves than larger larvae when reared at the same temperature (Hardy & Litvak, 2004); however, further studies would be beneficial to determine whether utilization efficiencies vary among different families.

### 4.5 Implications for sturgeon populations

Findings from our study have important management implications for the protection and propagation of sturgeon populations. First, results demonstrate that the mean and variability in temperatures experienced during egg incubation have considerable effects on larval body size and timing of development. Protection of stream rearing habitats requires maintaining natural thermal regimes from anthropogenic influences such as the release of pooled reservoir water from hydroelectric dams. Thermal variability can cause the release of cryptic genetic variation as observed in our study which may allow wild populations to produce adaptive phenotypes as environments change thereby tracking changes in adaptive fitness peaks; however, plastic responses to variable environments also have the potential to be nonadaptive by producing phenotypes that are distinct from the phenotypic optimum causing potential declines in population size (Ghalambor et al., 2015). Therefore, understanding whether plasticity facilitates or inhibits evolutionary responses of the population to environmental change is essential, particularly given the long generation time of sturgeon which limits their ability to quickly adapt to thermal changes.

Secondly, our results revealed that variation in body size and growth can differ between larvae reared under constant versus fluctuating conditions. If these size differences are persistent through the juvenile period, rearing individuals at constant water temperatures as opposed to ambient river water as done in stream‐side facilities (e.g., Crossman et al., 2011) could result in exaggerated differences in phenotypes and thus survival between hatchery‐produced and wild progeny. These potential size differentials should be considered in supplemental stocking and rehabilitation programs aimed toward increasing recruitment of naturally‐produced progeny in the population.

Results further show that genetic (family) effects have a considerable effect on phenotypic variation during early life stages. Larvae from families vary in size and growth even when experiencing the same rearing temperature. For propagation programs, these potential size differences among members of different genotypes should be considered to avoid artificial selection that could occur if stocking preferentially emphasized release of offspring of large body size. For the wild‐produced progeny, intra‐ and interannual variability in thermal conditions within a river may result in increased variability in phenotypic trait expression among individuals of differing genotype. Thus, selection may favor certain genotypes produced during different portions of the spawning season or in different years. At the population level, this differential survival has the potential to increase the relatedness among surviving individuals within a spawning season thereby increasing levels of coancestry (or identity‐by‐descent) within a year class. If selection continually favors the same genotypes across year classes, overall relatedness could also potentially increase leading to increased levels of coancestry among individuals from different year cohorts. Therefore, monitoring how thermal conditions affect traits associated with survival among genotypes is necessary for understanding potential changes to the genetic diversity of the population.

### 4.6 Conclusions and future directions

Our findings have important implications regarding the impact of predicted thermal changes due to climate change on phenotypic variation in ectothermic species. Understanding how variability in phenotypic trait expression due to the release of cryptic genetic variation is maintained across sequential ontogenetic stages in wild populations is vital to understanding how populations can respond to environmental change (Ledón‐Rettig et al., 2014). Additionally, reaction norms constructed from rearing individuals at constant temperatures are not reliable indicators of response under variable (and natural) conditions. Therefore, researchers may wish to construct “realized” thermal reaction norms by adding naturally occurring (e.g., diel) variability in temperature into experimental treatments (Paaijmans et al., 2013). Given our findings on phenotypic traits that are tied to survival, further empirical work addressing how incubation conditions and differences among families affect trait variation and survival at later ontogenetic stages would be beneficial to predict changes in the genetic composition of populations due to environmental perturbations. Knowledge within these research areas is essential to understand the ecological responses and evolutionary potential of a population to climate change.

## Data Arching Statement

Data files used for the analyses in this study are available at the Dryad Digital Repository: http://dx.doi.org/10.5061/dryad.pq35t.
