# Supplementary material for: Increases in the mean and variability of thermal regimes result in differential phenotypic responses among genotypes during early ontogenetic stages of lake sturgeon (Acipenser fulvescens)
Source: Evol Appl. 2016 Aug 31;9(10):1258–70. doi: 10.1111/eva.12409 (PMC5108217; doi:10.1111/eva.12409)
Supplement: Supplementary file 1 [file EVA-9-1258-s001.pdf]

Data S1

Title: Increases in the mean and variability of thermal regimes result in differential phenotypic responses among genotypes during early ontogenetic stages of lake sturgeon (*Acipenser fulvescens*)

Journal: Evolutionary Applications

Legend: Data S1 contains Density and Trace Plots (graphics) for the models of best fit for the three larval traits measured at hatch and three traits measured at emergence in the experiment. Models of best fit are provided where a (*) represents an interaction between variables. In all plots, Treatment A corresponds to the warm treatment, Treatment B corresponds to the variable treatment, Treatment C corresponds to the ambient treatment, and Treatment D corresponds to the cold treatment.

Hatch Traits

1. Body Length

Best Model: μ + IncubationTreatment + *HSFamily*IncubationTreatment* + $\varepsilon_{\text{heterogeneous}}$

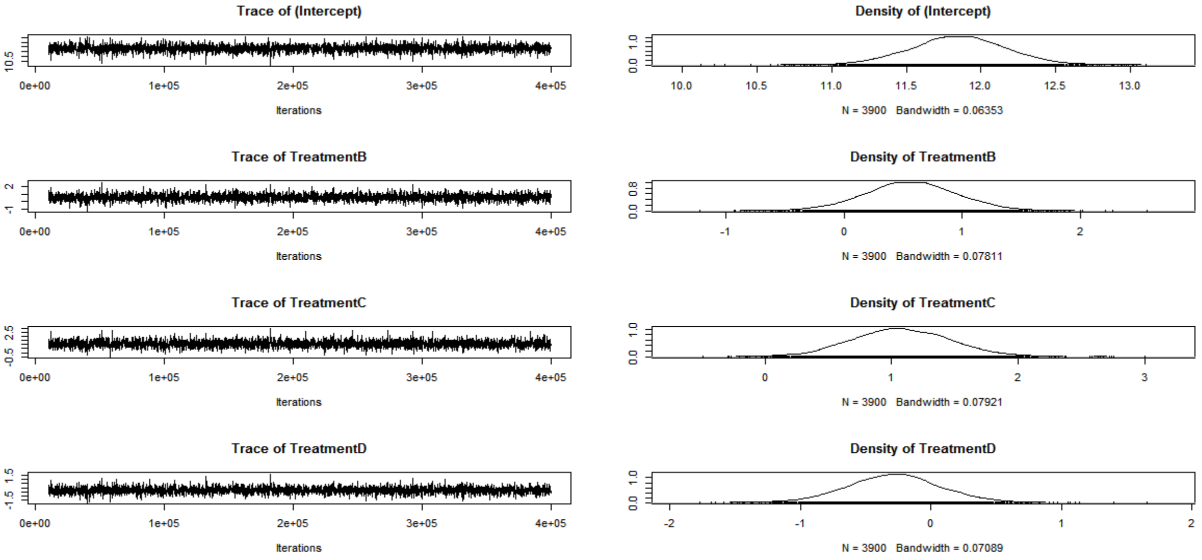

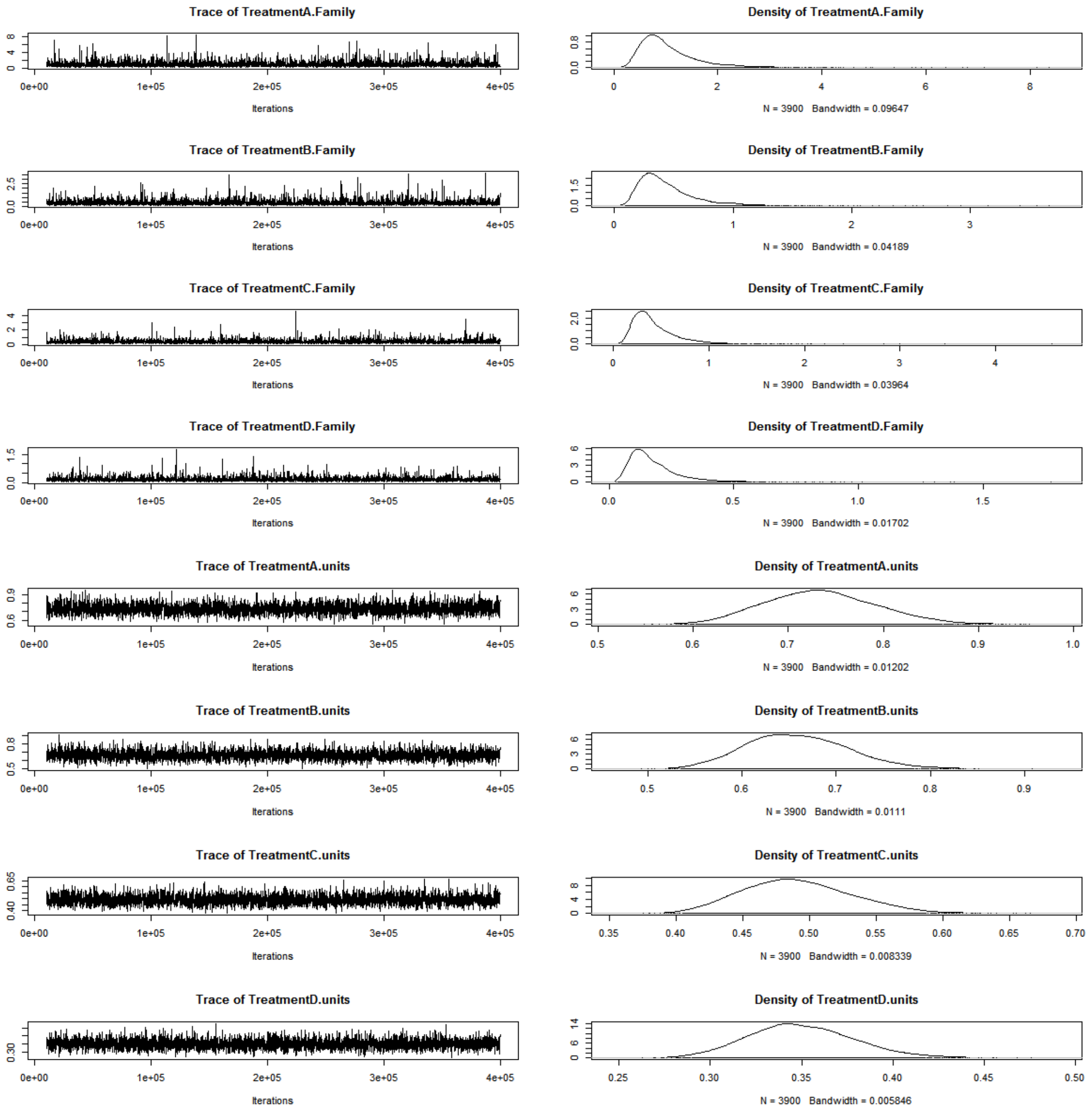
Trace of TreatmentA.Family
Density of TreatmentA.Family
Iterations
N = 3900 Bandwidth = 0.09647
Trace of TreatmentB.Family
Density of TreatmentB.Family
Iterations
N = 3900 Bandwidth = 0.04189
Trace of TreatmentC.Family
Density of TreatmentC.Family
Iterations
N = 3900 Bandwidth = 0.03964
Trace of TreatmentD.Family
Density of TreatmentD.Family
Iterations
N = 3900 Bandwidth = 0.01702
Trace of TreatmentA.units
Density of TreatmentA.units
Iterations
N = 3900 Bandwidth = 0.01202
Trace of TreatmentB.units
Density of TreatmentB.units
Iterations
N = 3900 Bandwidth = 0.0111
Trace of TreatmentC.units
Density of TreatmentC.units
Iterations
N = 3900 Bandwidth = 0.008339
Trace of TreatmentD.units
Density of TreatmentD.units
Iterations
N = 3900 Bandwidth = 0.005846

2. Body Area

Best Model: μ + IncubationTreatment + HSFamily*IncubationTreatment + $\varepsilon_{heterogeneous}$

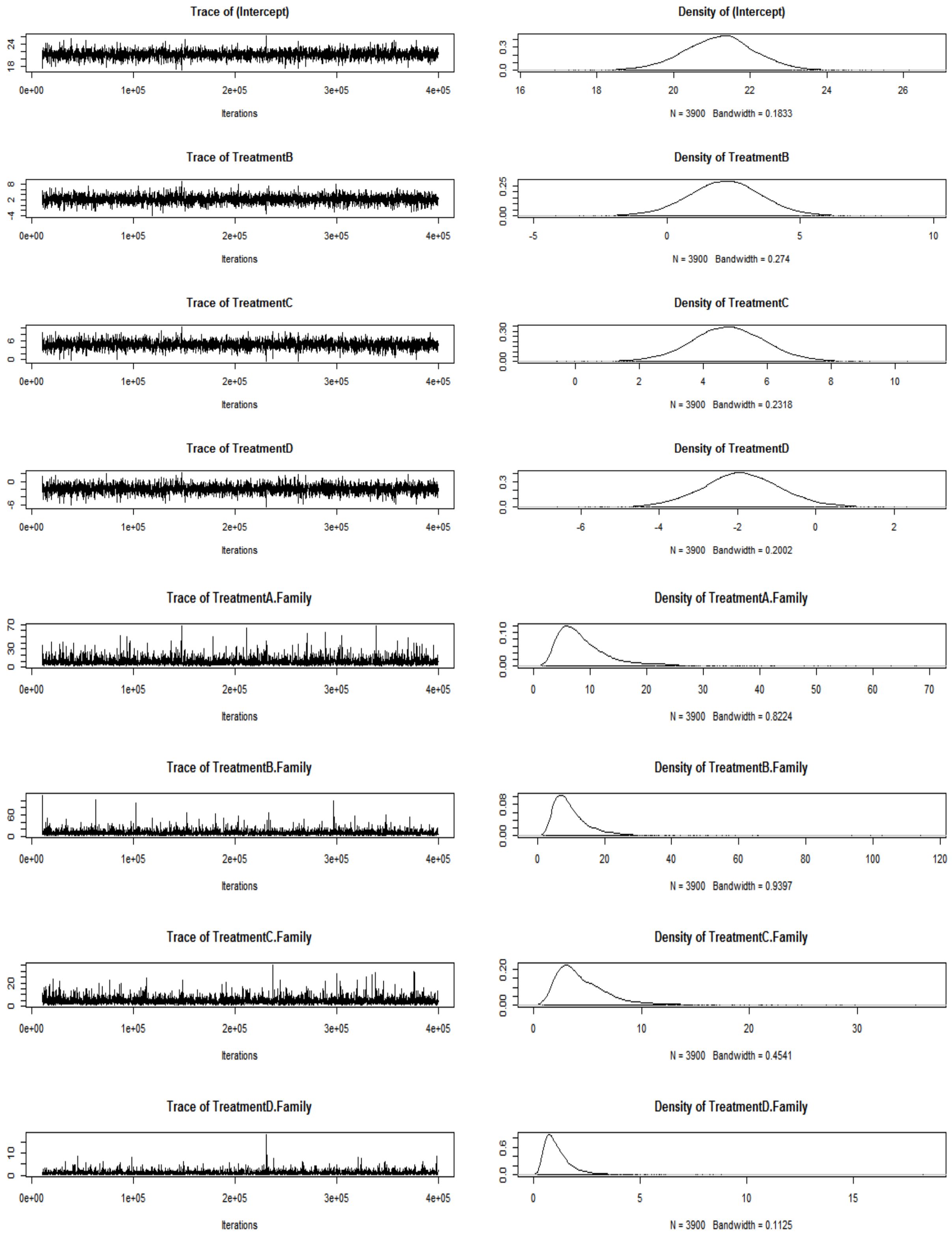

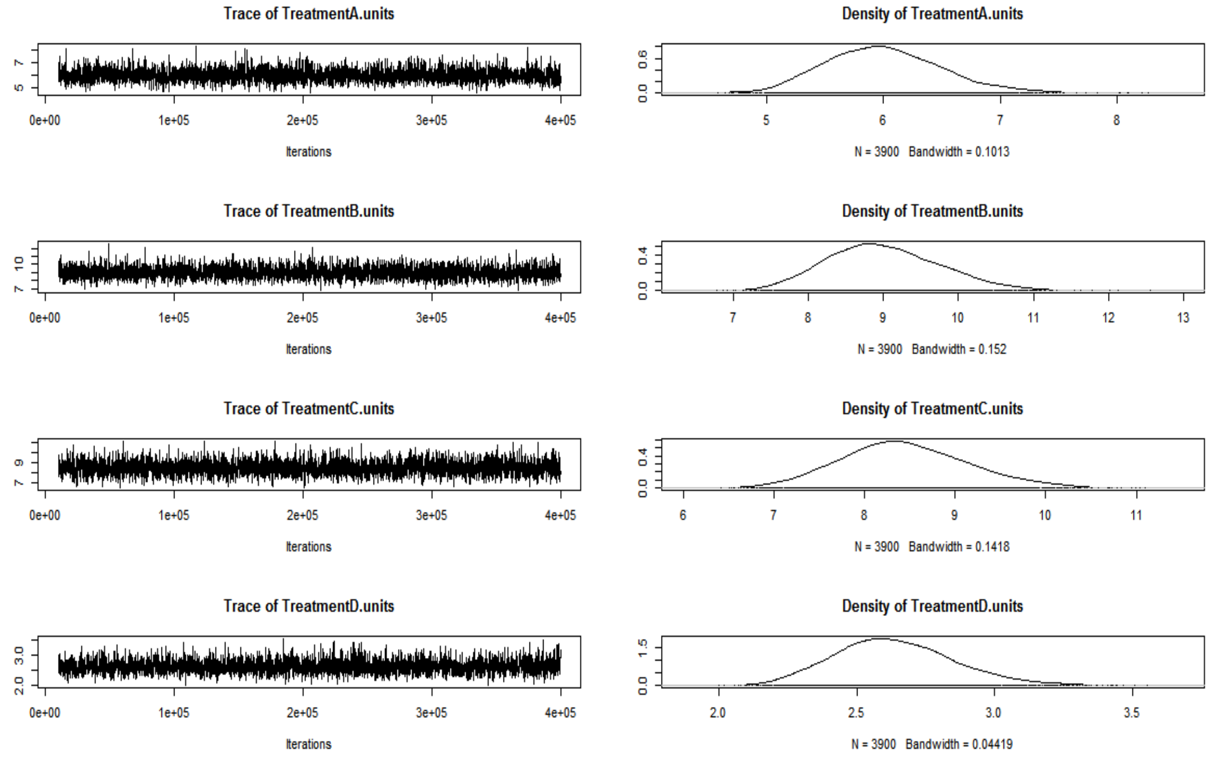

3. Yolk-Sac Area

Best Model: μ + IncubationTreatment + *HSFamily*IncubationTreatment* + $\varepsilon_{heterogeneous}$

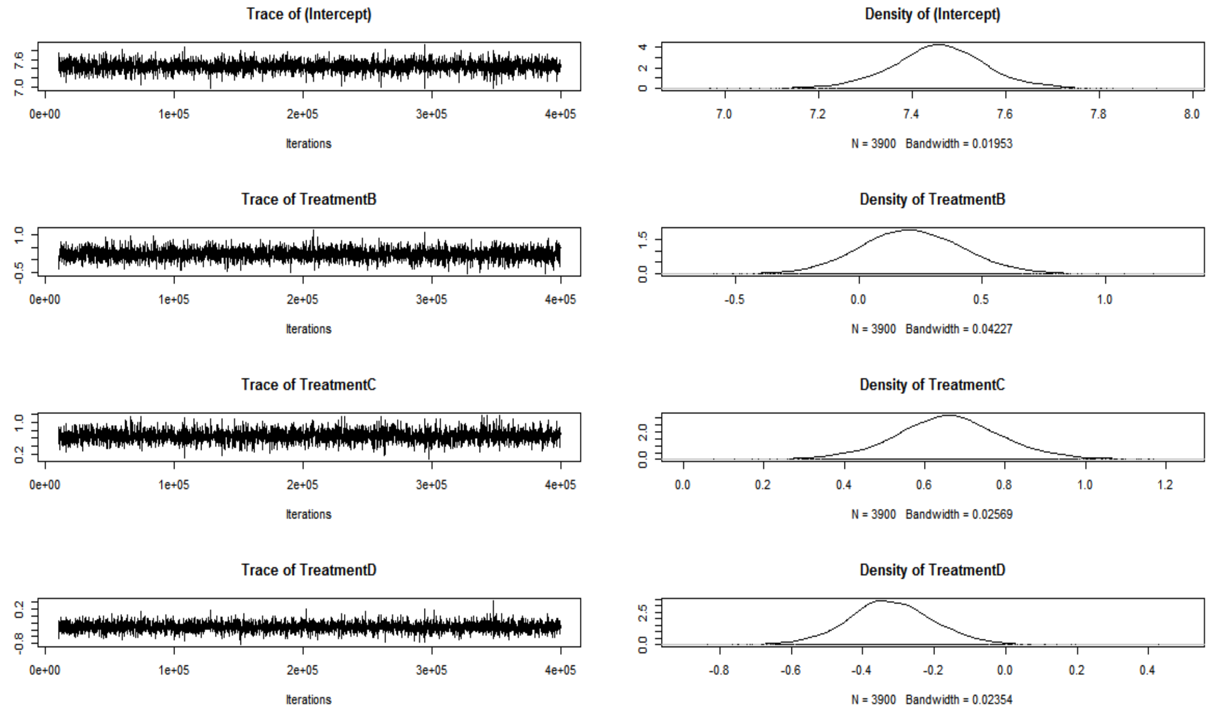

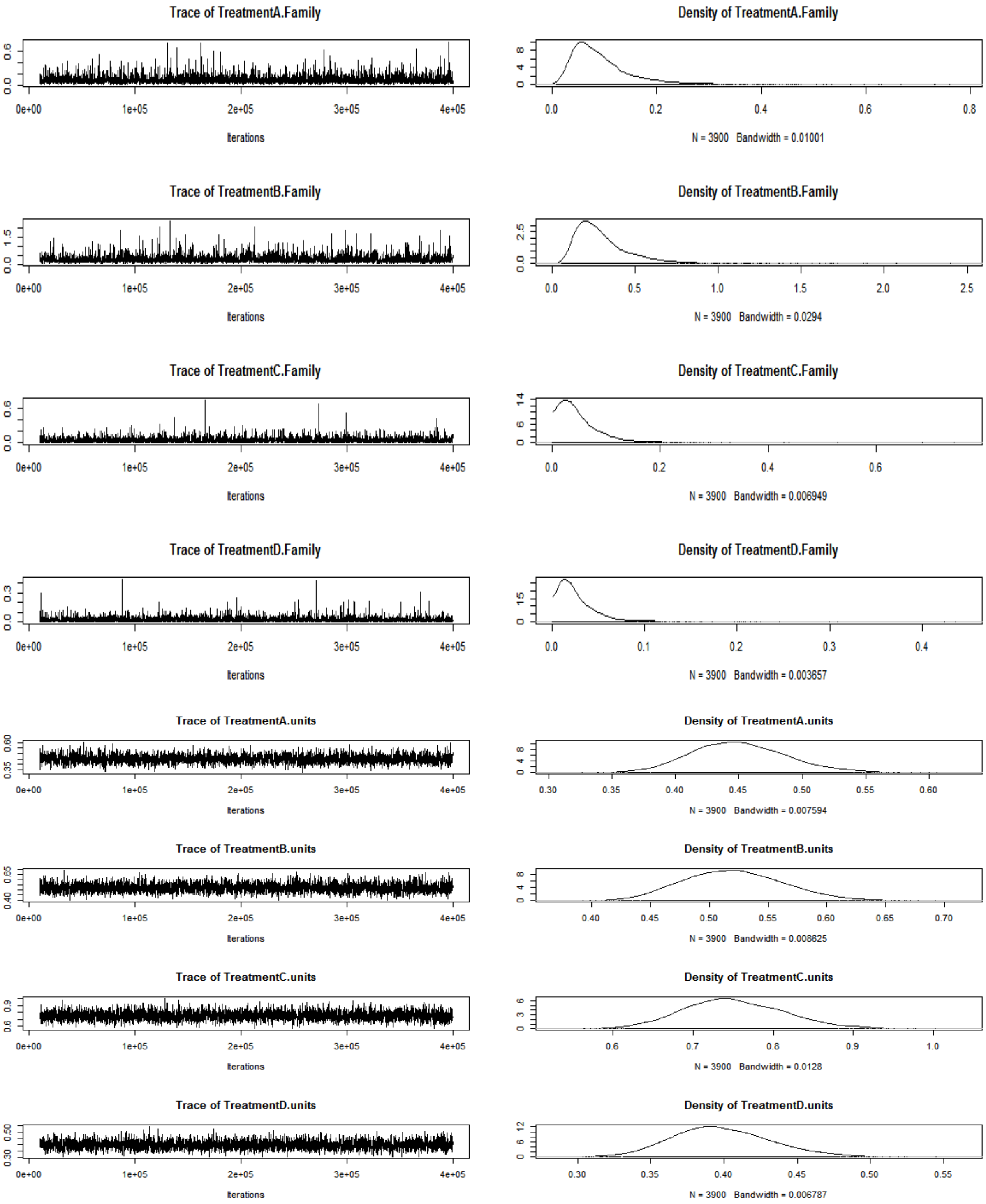
Trace of TreatmentA.Family
Iterations
Density of TreatmentA.Family
N = 3900 Bandwidth = 0.01001
Trace of TreatmentB.Family
Iterations
Density of TreatmentB.Family
N = 3900 Bandwidth = 0.0294
Trace of TreatmentC.Family
Iterations
Density of TreatmentC.Family
N = 3900 Bandwidth = 0.006949
Trace of TreatmentD.Family
Iterations
Density of TreatmentD.Family
N = 3900 Bandwidth = 0.003657
Trace of TreatmentA.units
Iterations
Density of TreatmentA.units
N = 3900 Bandwidth = 0.007594
Trace of TreatmentB.units
Iterations
Density of TreatmentB.units
N = 3900 Bandwidth = 0.008625
Trace of TreatmentC.units
Iterations
Density of TreatmentC.units
N = 3900 Bandwidth = 0.0128
Trace of TreatmentD.units
Iterations
Density of TreatmentD.units
N = 3900 Bandwidth = 0.006787

Emergence Traits

1. Time to Emergence

Best Model: μ + IncubationTreatment + DegreeDays + *FSFamily* + $\varepsilon_{homogeneous}$

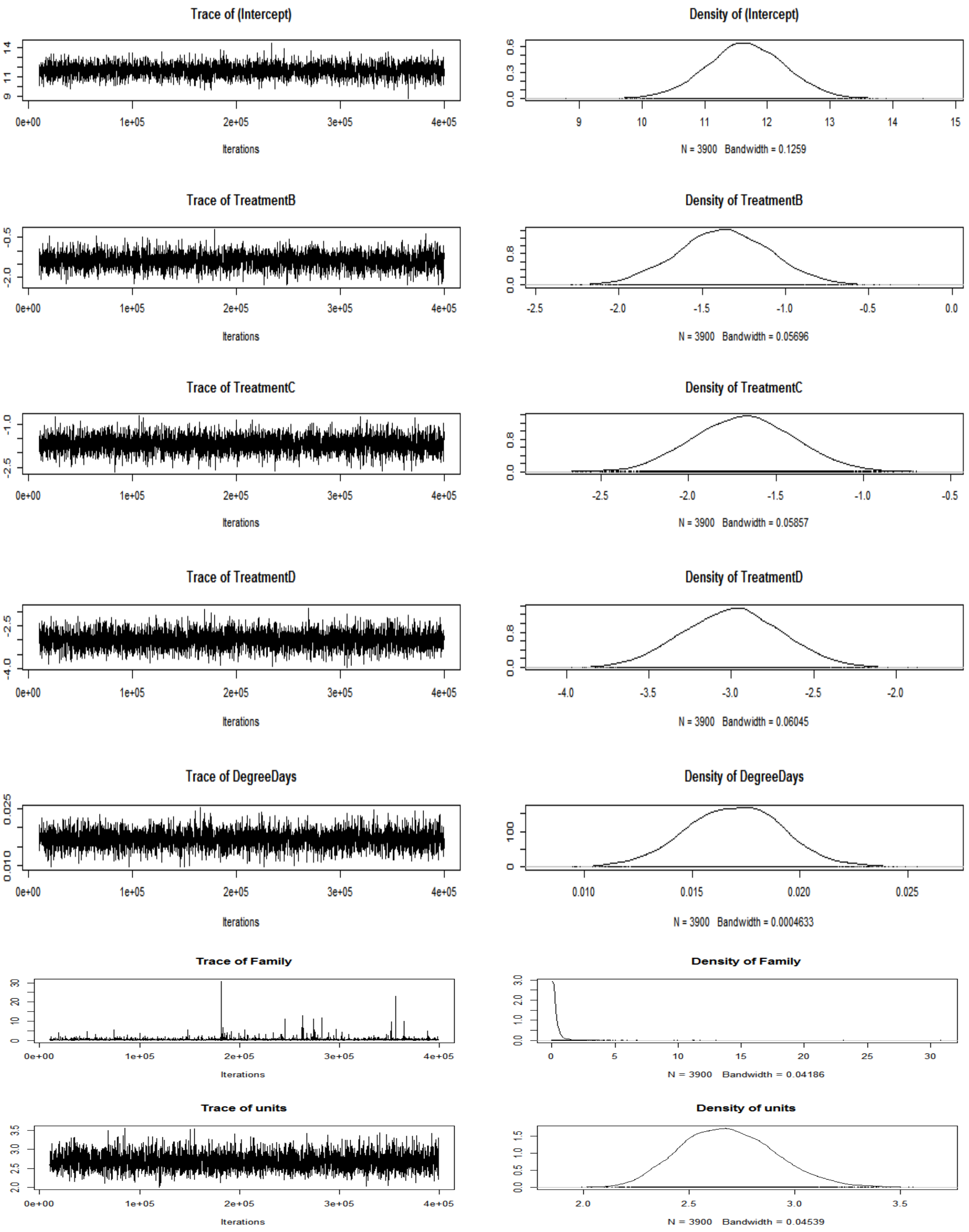

2. Emergence Body Length

Best model: (null) $\mu + \varepsilon_{homogeneous}$

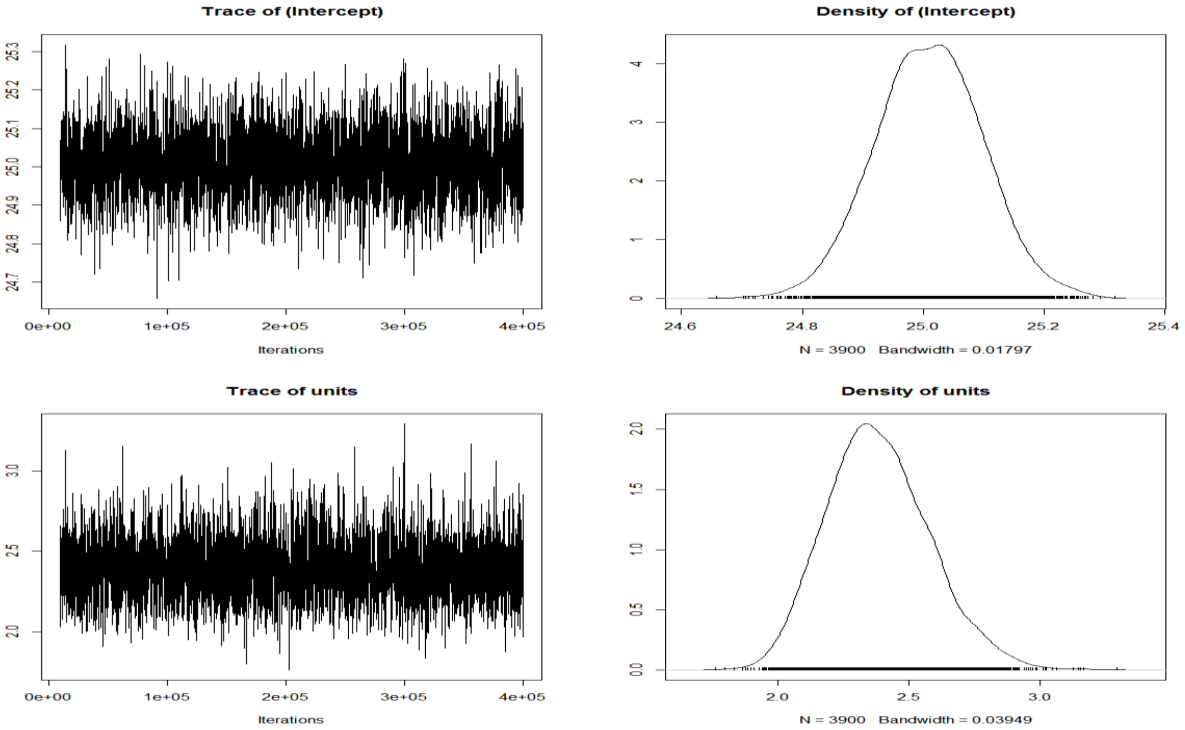

3. Total Growth

Best model: μ + IncubationTreatment + *FSFamily* + $\varepsilon_{homogeneous}$

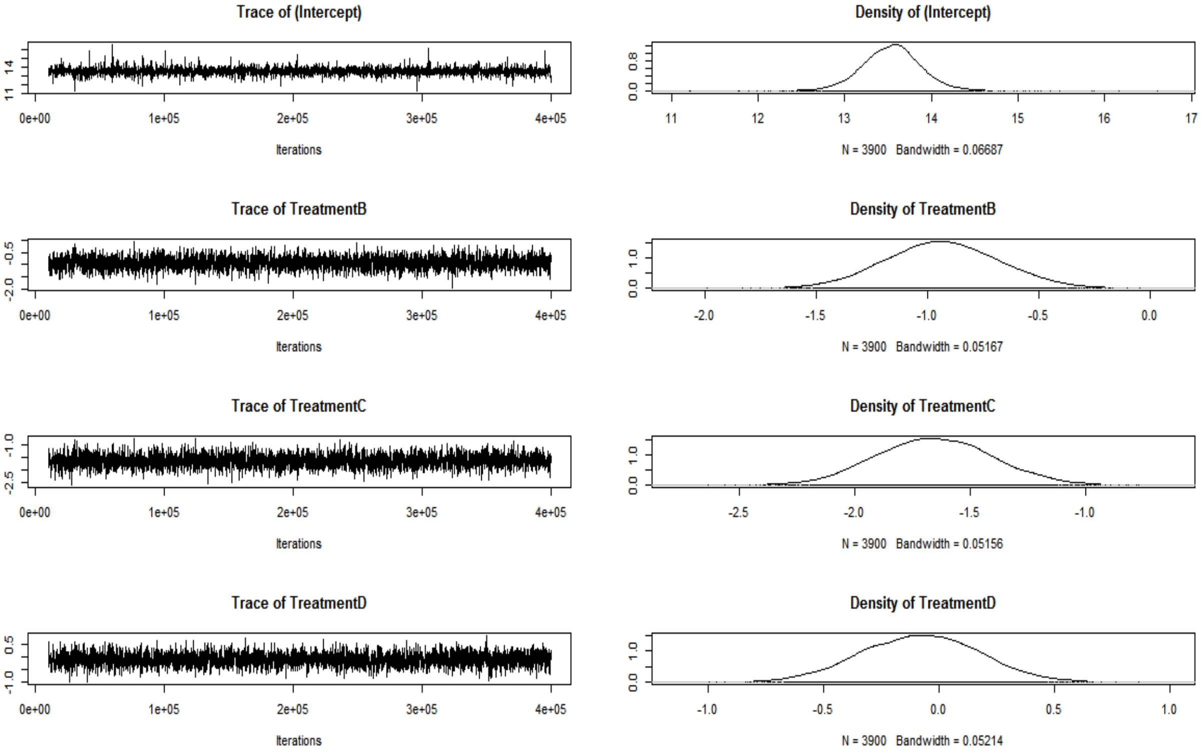

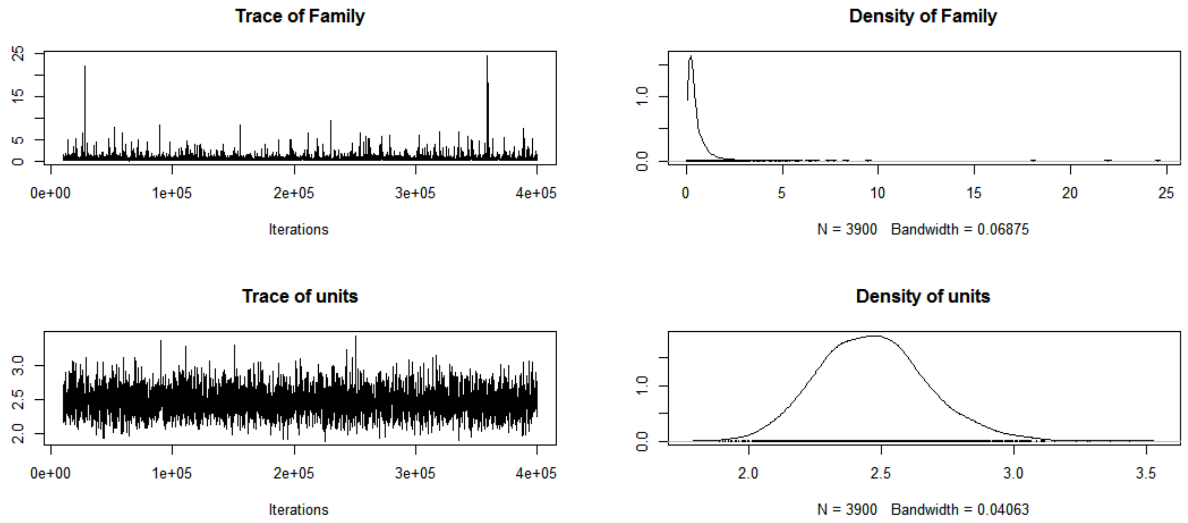
Trace of Family
Density of Family
Iterations
N = 3900   Bandwidth = 0.06875
Trace of units
Density of units
Iterations
N = 3900   Bandwidth = 0.04063
0e+00
1e+05
2e+05
3e+05
4e+05
0
5
10
15
20
25
0.0
1.0
2.0
2.5
3.0
3.5
