# Supplementary material for: Increases in the mean and variability of thermal regimes result in differential phenotypic responses among genotypes during early ontogenetic stages of lake sturgeon (Acipenser fulvescens)
Source: Evol Appl. 2016 Aug 31;9(10):1258–70. doi: 10.1111/eva.12409 (PMC5108217; doi:10.1111/eva.12409)
Supplement: Supplementary file 2 [file EVA-9-1258-s002.pdf]

Figs S1-S6

Title: Increases in the mean and variability of thermal regimes result in differential phenotypic responses among genotypes during early ontogenetic stages of lake sturgeon (*Acipenser fulvescens*)

Journal: Evolutionary Applications

Legend: Figs S1-S6 contains the thermal reaction norms plots for the three traits measured at hatch and three traits measured at the time of emergence for lake sturgeon larvae that were incubated in the four thermal treatments.

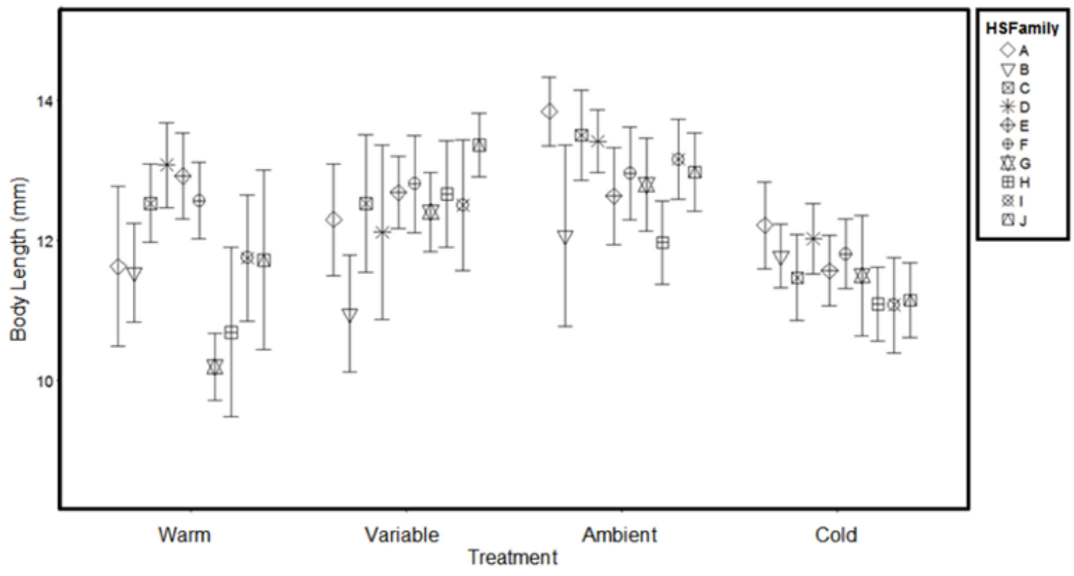

Figure S1. Thermal reaction norm plot including the means ± standard deviations for larval body length at hatch for 10 half-sibling (HS) lake sturgeon families reared in the four thermal treatments. Variation in body area among larvae was due to a HSFamily-by-IncubationTreatment interaction.

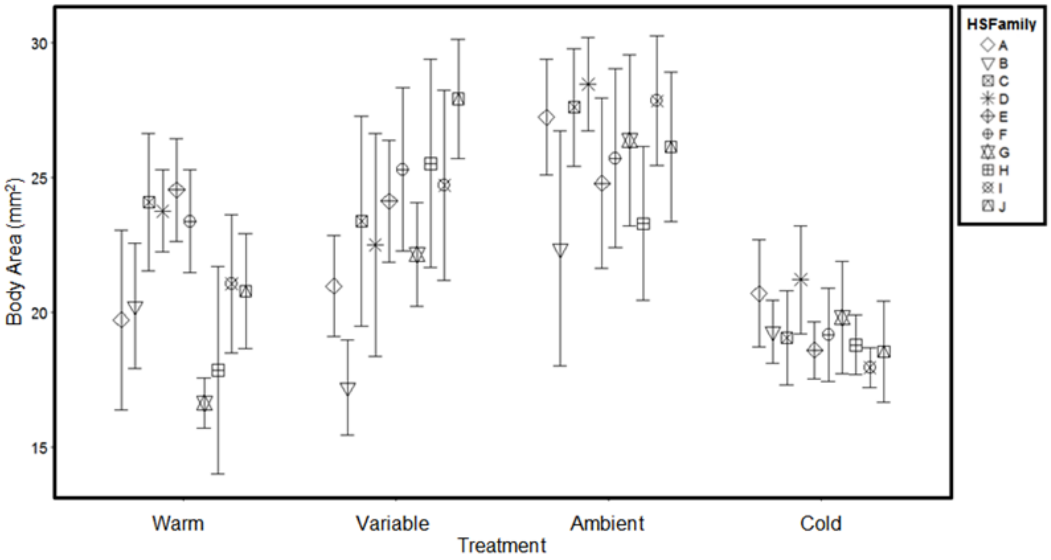

Figure S2. Thermal reaction norm plot including the means ± standard deviations for larval body area at hatch for 10 half-sibling (HS) lake sturgeon families reared in the four thermal treatments. Variation in body area among larvae was due to a HSFamily-by-IncubationTreatment interaction.

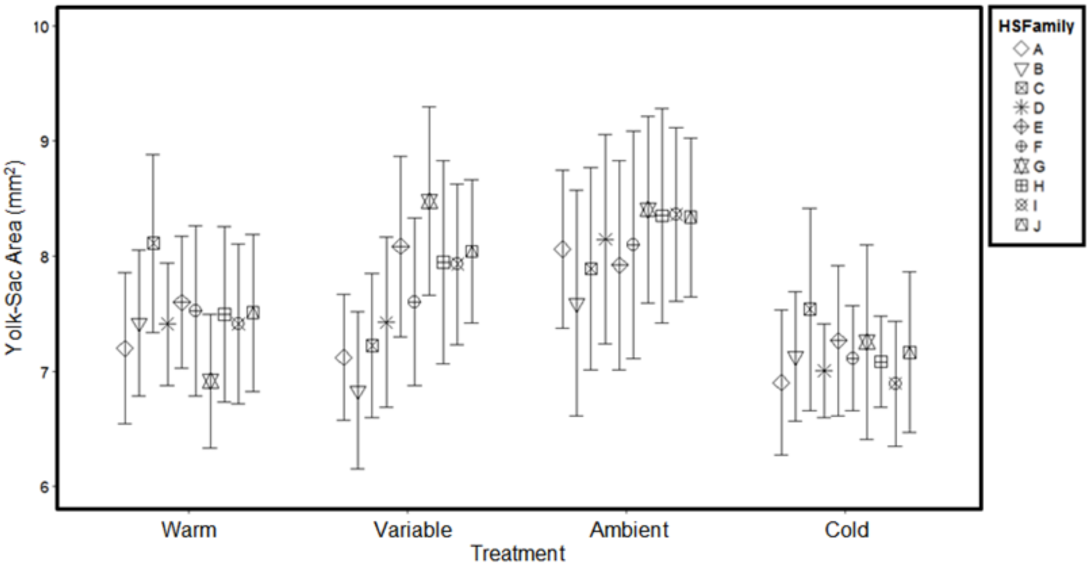

Figure S3. Thermal reaction norm plot including the means ± standard deviations for larval yolk-sac area at hatch for 10 half-sibling (HS) lake sturgeon families reared in the four thermal treatments. Variation in yolk-sac area among larvae was due to a HSFamily-by-IncubationTreatment interaction.

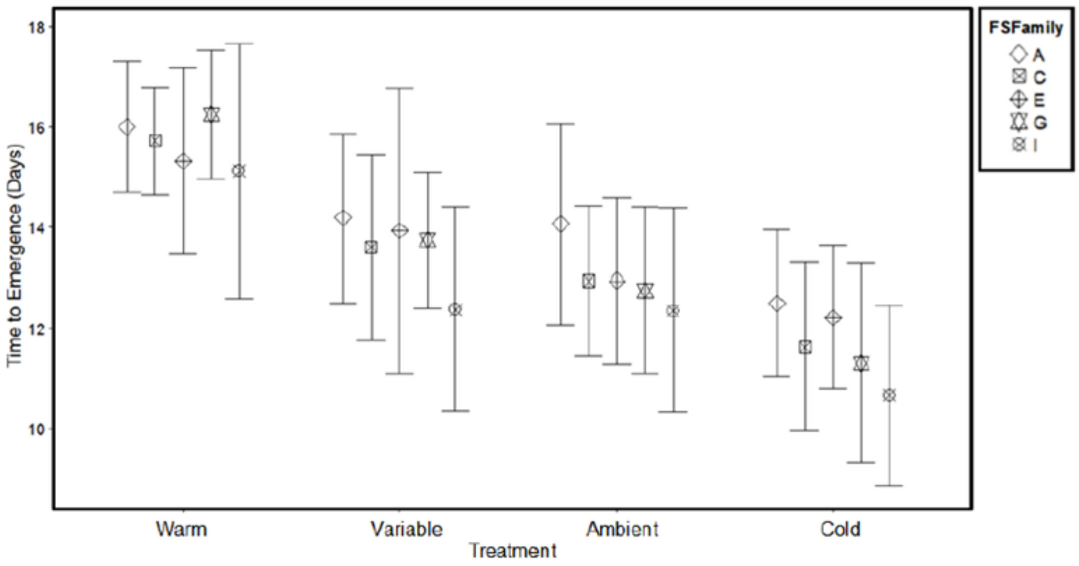

Figure S4. Thermal reaction norm plot including the means ± standard deviations for the timing of emergence for the 5 full-sibling (FS) lake sturgeon families that incubated in the four thermal treatments before being monitored until emergence. Variation in the timing of emergence among larvae was due to a FS Family effect.

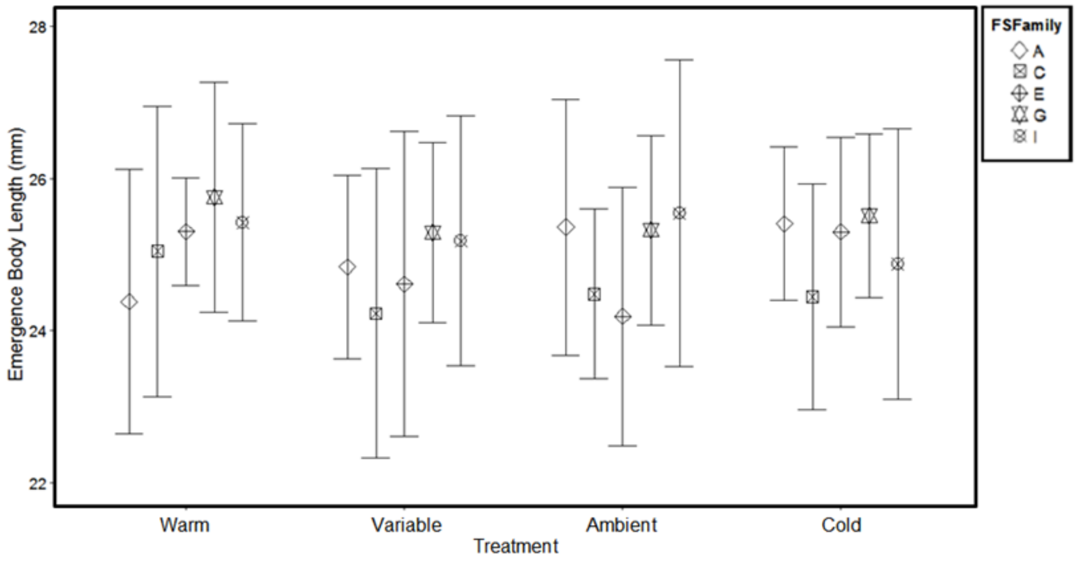

Figure S5. Thermal reaction norm plot including the means ± standard deviations for body length at the time of emergence for 5 full-sibling (FS) lake sturgeon families that incubated in the four thermal treatments and were monitored until emergence. Thermal incubation environment and parentage had no effect on larval body length at the time of emergence.

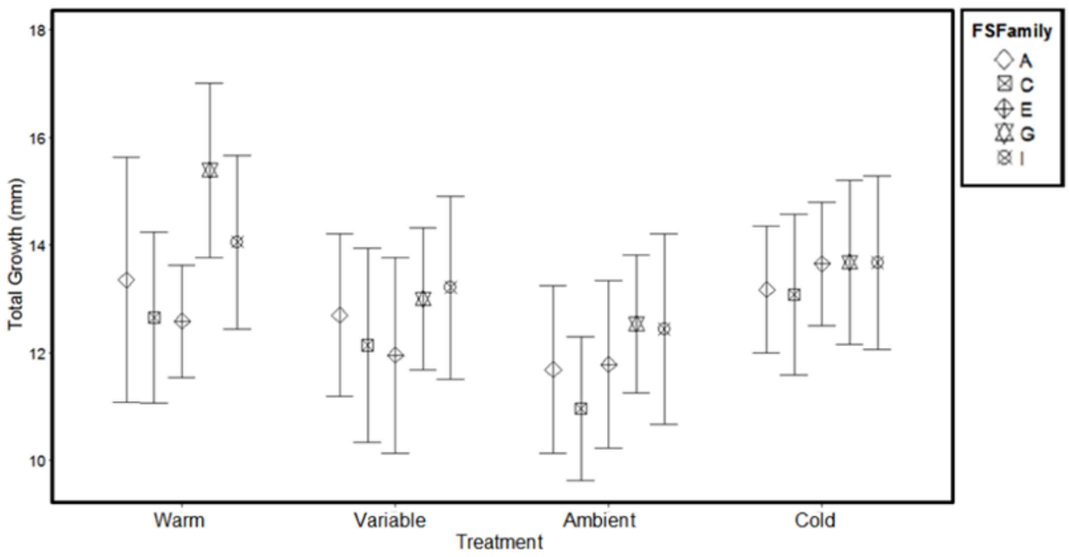

Figure S6. Thermal reaction norm plot including the means ± standard deviations for total growth from hatch to emergence for the 5 full-sibling (FS) lake sturgeon families which incubated in the four thermal treatments before monitoring until emergence. There was an effect of FS Family on growth.
