# Supplementary material for: Increases in the mean and variability of thermal regimes result in differential phenotypic responses among genotypes during early ontogenetic stages of lake sturgeon (Acipenser fulvescens)
Source: Evol Appl. 2016 Aug 31;9(10):1258–70. doi: 10.1111/eva.12409 (PMC5108217; doi:10.1111/eva.12409)
Supplement: Supplementary file 3 [file EVA-9-1258-s003.pdf]

Table S1

Title: Increases in the mean and variability of thermal regimes result in differential phenotypic responses among genotypes during early ontogenetic stages of lake sturgeon (*Acipenser fulvescens*)

Journal: Evolutionary Applications

Legend: Table S1 contains a table of DIC values used in conjunction with model diagnostics (Data S1) to determine the model of best fit for the three traits measured at hatch and three traits measured at emergence.

Table S1. Step-wise model comparisons and DIC-values used in conjunction with diagnostic plots to determine the best model for the three larval traits quantified at hatch (a) and three traits quantified at the time of emergence (b) in the experiment. In the traits measured at hatch, family covariances were approximately zero suggesting removal of the main effect of HSFamily despite the presence of an interaction would be an adequate model. Models included heterogeneous residual variances (ε) except when compared against simpler models with homogeneous residual variances ($\varepsilon_{homogeneous}$) where applicable to determine the best overall model (*).

Larval Traits	Model	DIC
(a) Measured at Hatch		
Body Length	μ + IncubationTreatment + *HSFamily* + *HSFamily*IncubationTreatment* + ε	2698.367
	μ + IncubationTreatment + *HSFamily*IncubationTreatment* + ε	2698.346*
	μ + IncubationTreatment + *HSFamily* + ε	3066.084
	μ + IncubationTreatment + ε	3241.379
	μ + ε	3637.882
Body Area	μ + IncubationTreatment + *HSFamily* + *HSFamily*IncubationTreatment* + ε	5571.782
	μ + IncubationTreatment + *HSFamily*IncubationTreatment* + ε	5571.753*
	μ + IncubationTreatment + *HSFamily* + ε	6038.554
	μ + IncubationTreatment + ε	6124.094
	μ + ε	6821.935
Yolk-sac Area	μ + IncubationTreatment + *HSFamily* + *HSFamily*IncubationTreatment* + ε	2639.949
	μ + IncubationTreatment + *HSFamily*IncubationTreatment* + ε	2640.882*
	μ + IncubationTreatment + *HSFamily* + ε	2753.295
	μ + IncubationTreatment + ε	2790.816
	μ + ε	3045.075
(b) Measured at Time of Emergence		
Time to Emergence	μ + IncubationTreatment + MeanT + DegreeDays + Raceway + *FSFamily* + *FSFamily*IncubationTreatment* + ε	1167.853
	μ + IncubationTreatment + MeanT + DegreeDays + Raceway + *FSFamily* + ε	1169.618
	μ + IncubationTreatment + MeanT + DegreeDays + *FSFamily* + ε	1165.823
	μ + IncubationTreatment + DegreeDays + *FSFamily* + ε	1164.117

	μ + IncubationTreatment + DegreeDays + *FSFamily* + $\varepsilon_{homogeneous}$	1165.728*
	μ + IncubationTreatment + *FSFamily* + ε	1221.246
	μ + IncubationTreatment + ε	1236.194
	$\mu + \varepsilon$	1372.526
Emergence Body Length	μ + IncubationTreatment + MeanT + DegreeDays + Raceway + *FSFamily* + *FSFamily*IncubationTreatment* + ε	1134.390
	μ + IncubationTreatment + MeanT + DegreeDays + Raceway + *FSFamily* + ε	1133.508
	μ + IncubationTreatment + MeanT + DegreeDays + *FSFamily* + ε	1129.972
	μ + IncubationTreatment + DegreeDays + *FSFamily* + ε	1127.939
	μ + IncubationTreatment + *FSFamily* + ε	1127.984
	μ + IncubationTreatment + ε	1132.060
	μ + IncubationTreatment + $\varepsilon_{homogeneous}$	1128.526
	$\mu + \varepsilon_{homogeneous}$	1124.330*
Total Growth	μ + IncubationTreatment + MeanT + DegreeDays + Raceway + *FSFamily* + *FSFamily*IncubationTreatment* + ε	1148.035
	μ + IncubationTreatment + MeanT + DegreeDays + Raceway + *FSFamily* + ε	1148.457
	μ + IncubationTreatment + MeanT + DegreeDays + *FSFamily* + ε	1145.658
	μ + IncubationTreatment + DegreeDays + *Family* + ε	1143.790
	μ + IncubationTreatment + *FSFamily* + ε	1144.507
	μ + IncubationTreatment + *FSFamily* + $\varepsilon_{homogeneous}$	1141.894*
	μ + IncubationTreatment + $\varepsilon_{homogeneous}$	1166.665
	$\mu + \varepsilon_{homogeneous}$	1207.014
