# Supplementary material for: Increases in the mean and variability of thermal regimes result in differential phenotypic responses among genotypes during early ontogenetic stages of lake sturgeon (Acipenser fulvescens)
Source: Evol Appl. 2016 Aug 31;9(10):1258–70. doi: 10.1111/eva.12409 (PMC5108217; doi:10.1111/eva.12409)
Supplement: Supplementary file 4 [file EVA-9-1258-s004.pdf]

Table S2

Title: Increases in the mean and variability of thermal regimes result in differential phenotypic responses among genotypes during early ontogenetic stages of lake sturgeon (*Acipenser fulvescens*)

Journal: Evolutionary Applications

Legend: Table S2 contains a table of the Fixed effect model outputs from the models of best fit for the three larval traits measured at hatch and three traits measured at the time of emergence in the experiment.

Table S2. Outputs for the fixed effects from the models of best fit for the three traits measured at hatch (a) and three traits measured at the time of emergence (b). Parameters were estimated as the mode of the posterior distribution ± the Highest Posterior Density (HPD). The pMCMC (a measure of the tail area probability of the posterior density) output provided by MCMCglmm is reported.

Phenotypic Traits	Component	Posterior mode	±95% HPD	pMCMC
(a) Measured at Hatch				
Body Length	Intercept	11.89	(11.20, 12.50)	<0.001*
	Variable	0.51	(0.56, 0.77)	0.147
	Ambient	1.16	(0.37, 1.92)	0.007*
	Cold	-0.45	(-0.99, 0.42)	0.406
Body Area	Intercept	21.19	(19.23, 23.05)	<0.001*
	Variable	2.47	(-0.72, 4.97)	0.120
	Ambient	4.80	(2.30, 7.07)	<0.002*
	Cold	-1.81	(-3.92, 0.22)	0.070
Yolk-sac Area	Intercept	7.46	(7.25, 7.67)	<0.001*
	Variable	0.15	(-0.19, 0.65)	0.304
	Ambient	0.67	(0.38, 0.93)	<0.001*
	Cold	-0.31	(-0.56, -0.07)	0.014*
(b) Measured at Time of Emergence				
Time to Emergence	Intercept	11.43	(10.09, 12.60)	<0.001*
	Variable	-1.41	(-1.89, -0.75)	<0.001*
	Ambient	-1.58	(-2.19, -1.02)	<0.001*
	Cold	-2.94	(-3.45, -2.39)	<0.001*
	DegreeDays	0.02	(0.01, 0.02)	<0.001*
Emergence Body Length	Intercept	25.00	(24.83, 25.18)	<0.001*
Total Growth	Intercept	13.54	(12.78, 14.29)	<0.001*
	Variable	-1.03	(-1.44, -0.45)	<0.001*
	Ambient	-1.68	(-2.18, -1.17)	<0.001*
	Cold	-0.07	(-0.55, 0.44)	0.756

*Indicates a pMCMC<0.05
